# Supplementary material for: Evolution of core archetypal phenotypes in progressive high grade serous ovarian cancer
Source: Nat Commun. 2021 May 24;12:3039. doi: 10.1038/s41467-021-23171-3 (PMC8144406; doi:10.1038/s41467-021-23171-3)
Supplement: Supplementary file 1 — Supplementary Information [file 41467_2021_23171_MOESM1_ESM.pdf]

Supplementary Figure 1: Longitudinal cohort clusters before batch-correction

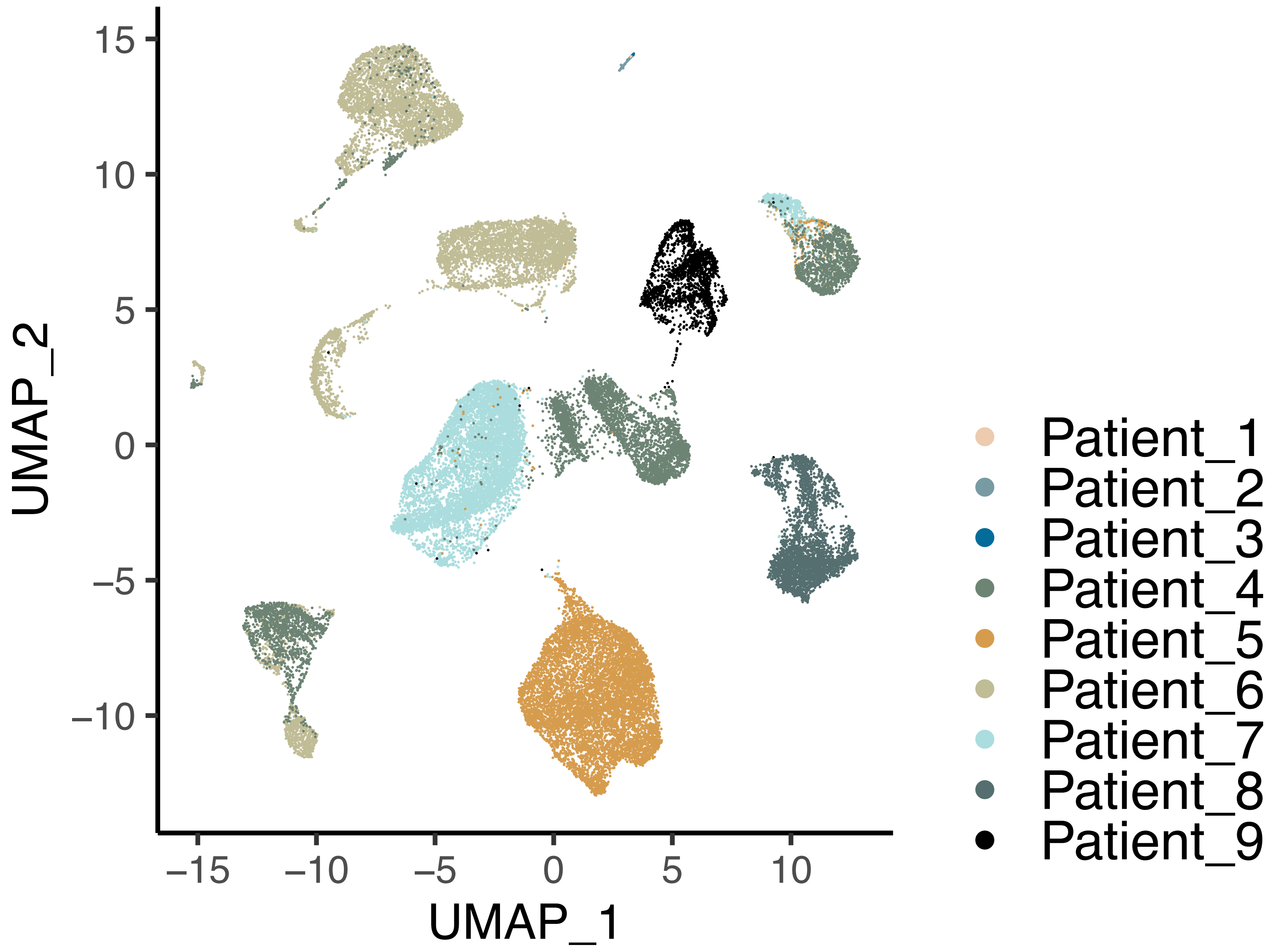

UMAP projection of scRNA-seq profiles of longitudinal HGSOC cohort (patients 1-9) across all time points prior to batch-correction. The cells are colored according to patient number.

Supplementary Figure 2: Expression levels of individual cell type identification markers

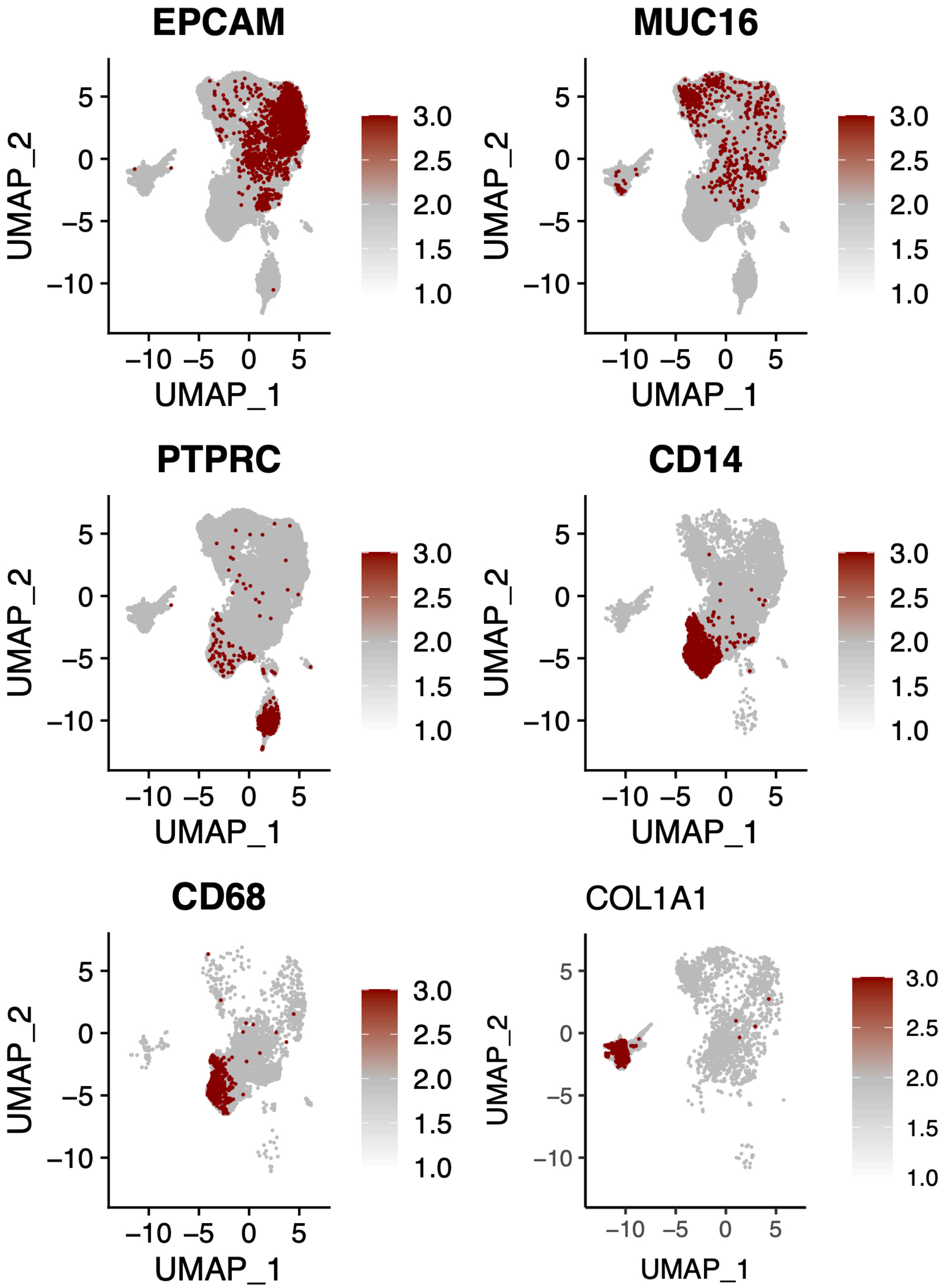

Supplementary Figure 3: Overlap of differentially expressed genes over time

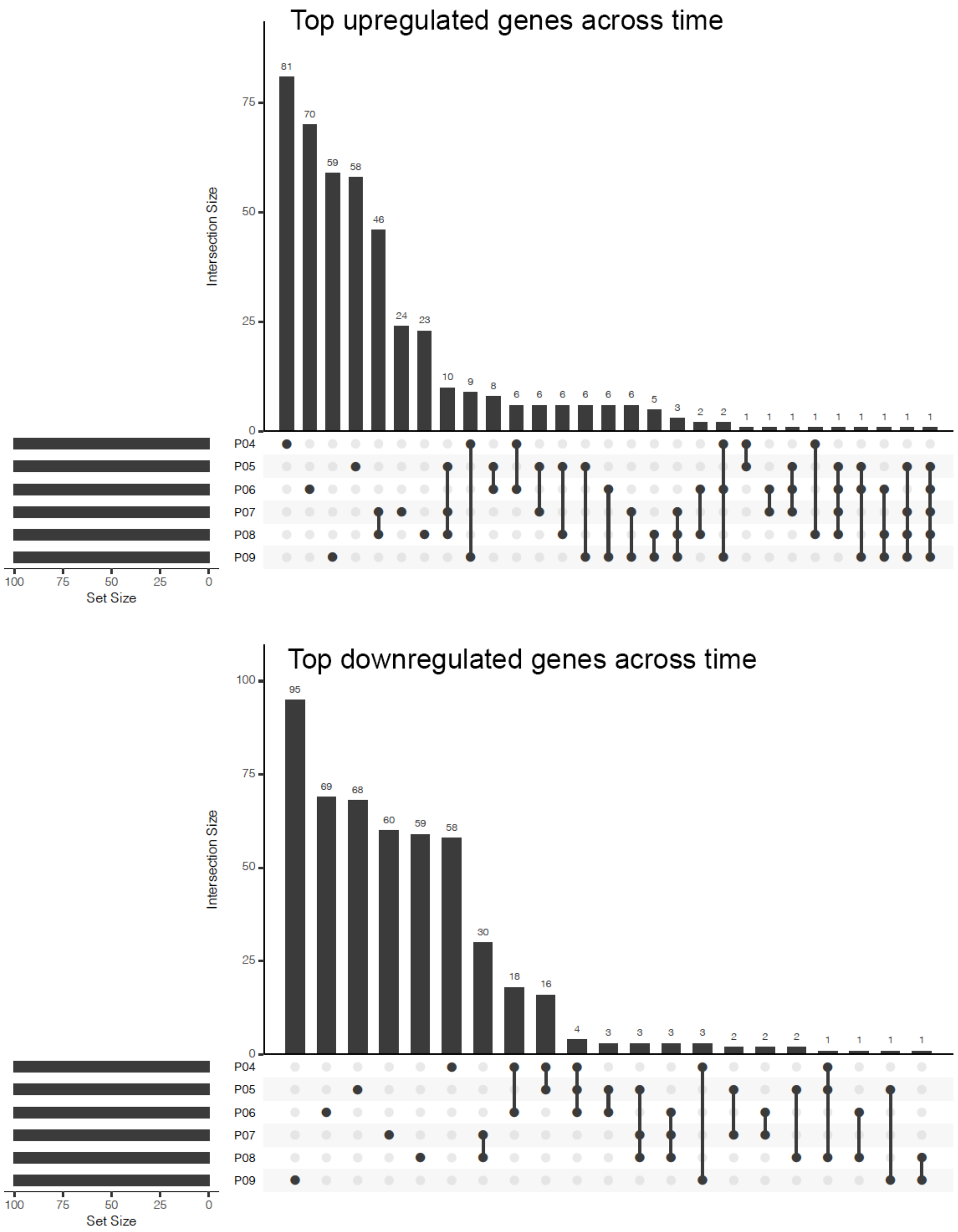

Upset plots displaying the frequency of unique and overlapping differentially expressed genes between the patients. The top panel shows the overlap of the top 100 upregulated genes over time while the bottom panel shows top 100 down regulated genes.

Supplementary Figure 4: Selecting optimal number of archetypes to enclose integrated longitudinal cohort scRNA-seq data

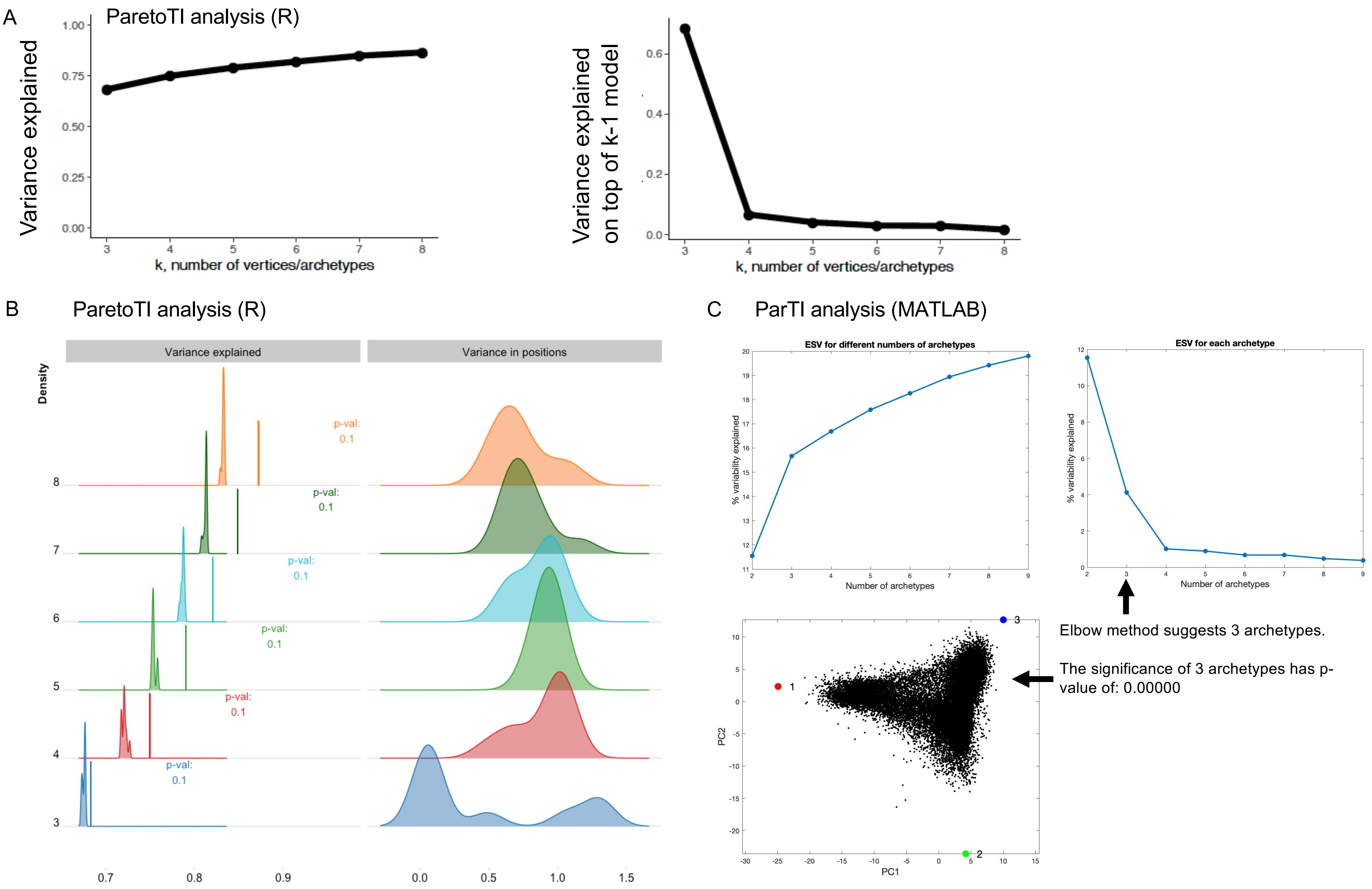

A. Variance explained by 3-8 archetypes (left panel) and gain in variance explained in by adding archetypes (right panel) B. Density plots of variance explained by 3-8 archetypes (left panel) and variance in position of the archetypes (right panel). P-values indicate empirical probabilities of variance explained by the data as a weighted sum of archetypes across 1000 randomizations C. Confirmation of archetype number using ParTI analysis of integrated counts data (top panel) and confirmation that a 3-vertex polytope (bottom panel) is a significant fit for the data based on a t-ratio test (one-sided). The t-ratio is the ratio of the volume of the polytope to the volume of the convex hull of the data. The P-value of the t-ratio test indicates the proportion of randomized sets with a t-ratio smaller than or equal to that of the original data.

Supplementary Figure 5: Multi-task model cross-validation analyses

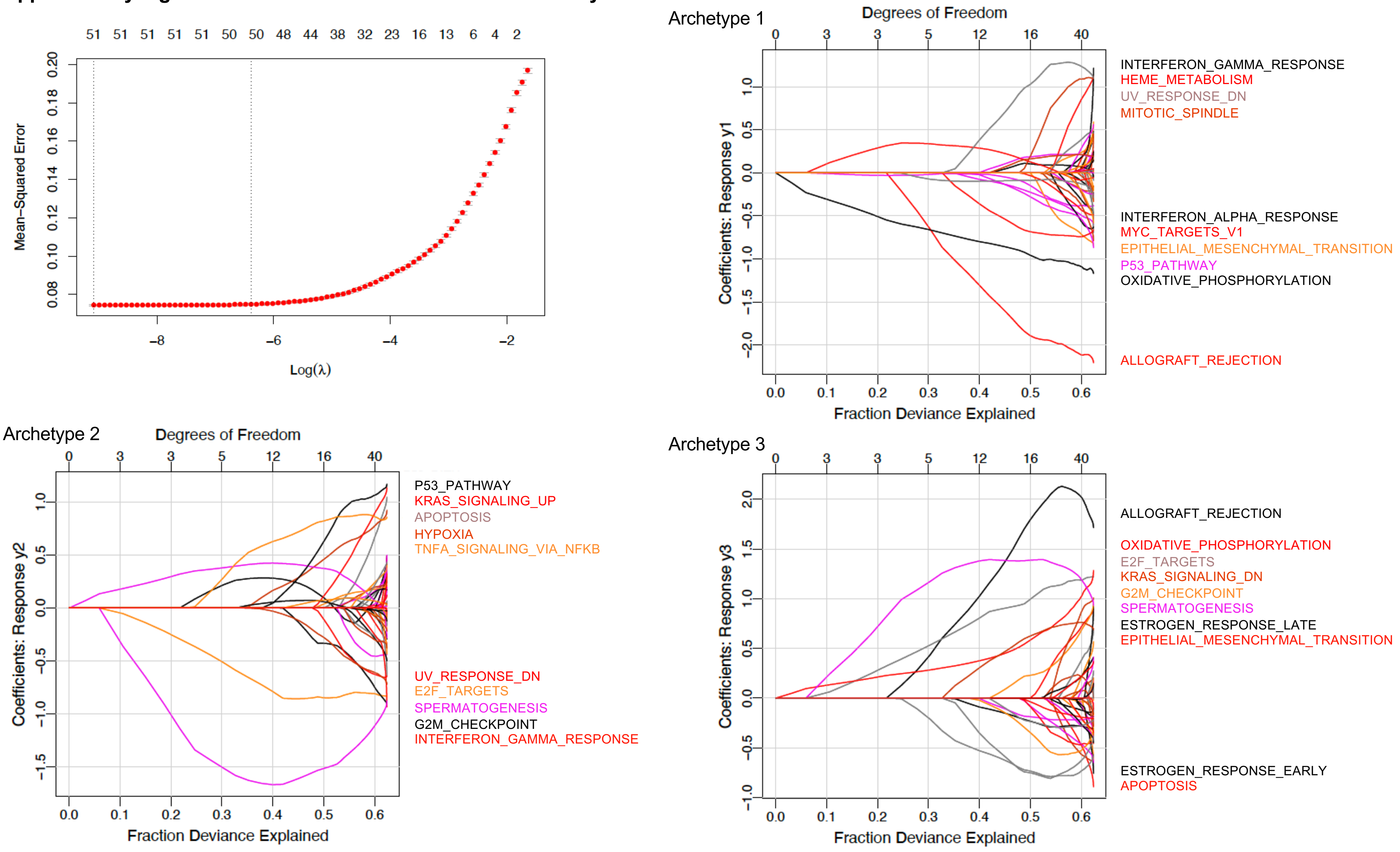

The top left panel displays mean squared error from the 10-fold cross-validation analysis of the group-lasso models with varying penalty (lambda), with number of features (pathways) in the model listed on top. The model was trained against 27997 archetype scores for each archetype across the nine patient samples with ssGSEA scores from 50 hallmark pathways. The three subsequent panels show the fraction of variance in the archetypes scores explained by the top hallmark pathway features for each of the three archetypes.

Supplementary Figure 6: Hierarchical clustering of hallmark pathway coefficients

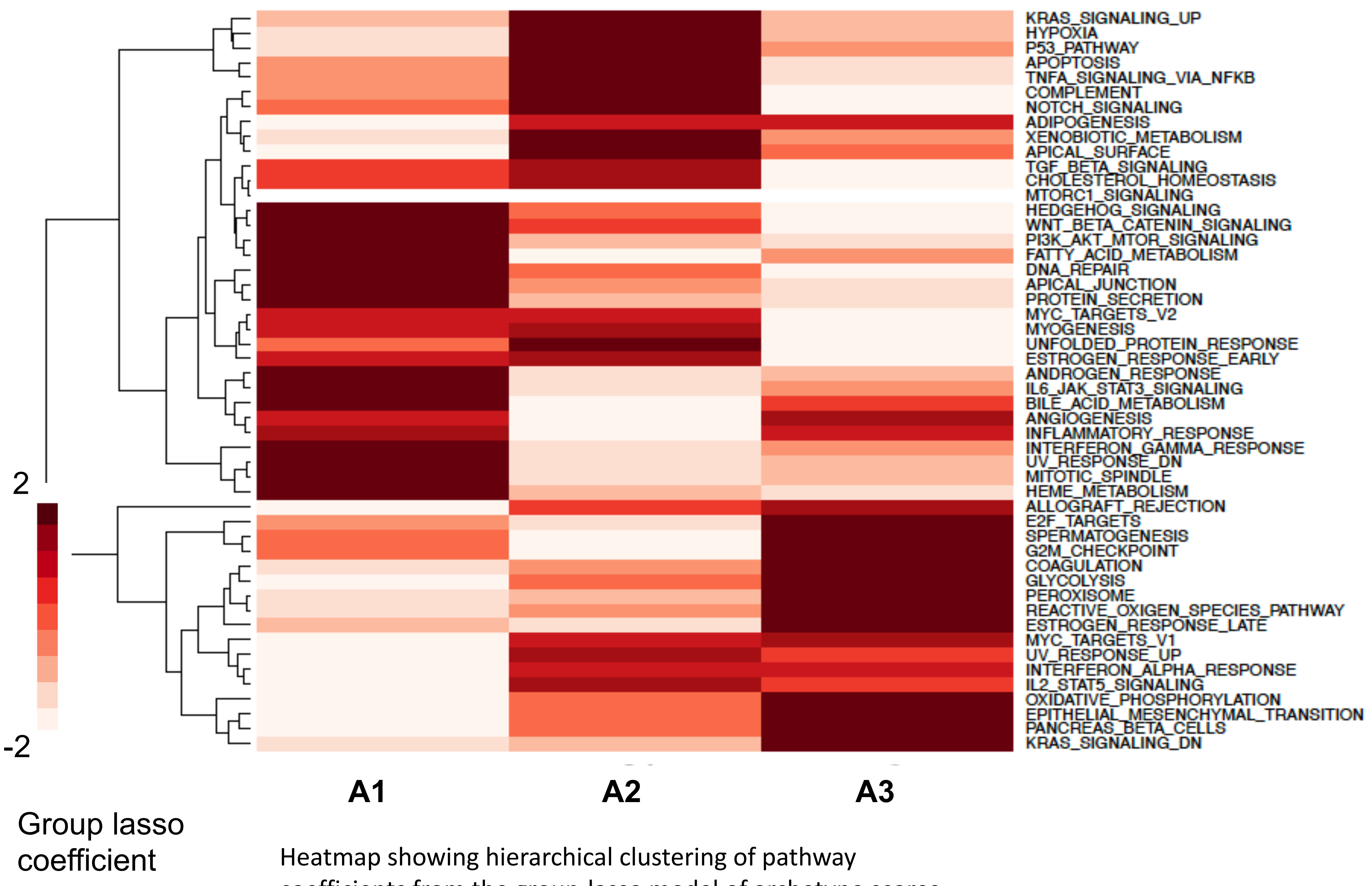

Heatmap showing hierarchical clustering of pathway coefficients from the group-lasso model of archetype scores

Supplementary Figure 7: Key pathway contributors of the MAP archetype

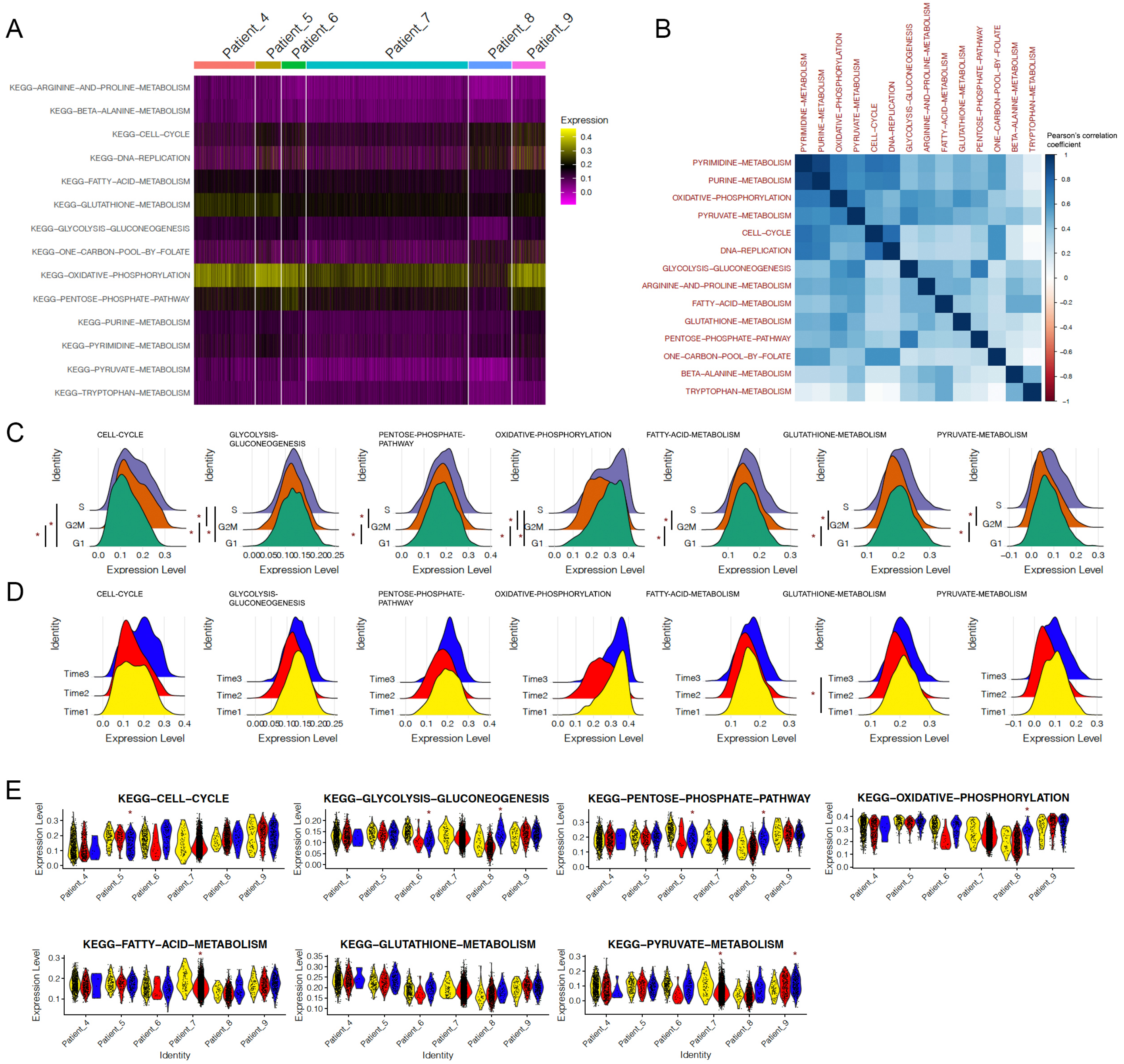

A. Heatmap displaying the gene set enrichment scores of key metabolism and proliferation KEGG pathways across single cells classified as MAP specialists. B. Correlation plot of key metabolism and proliferation KEGG pathways. The colors indicate magnitude of Pearson's correlation between the enrichment scores. C-D Ridge plots showing distribution of pathway enrichment scores across phases of cell cycle (C) or time (D). The vertical bars annotated with * show pairwise comparisons that are statistically significant from Tukey HSD test (one-sided) following ANOVA. E. Violin plots showing comparison of pathway enrichment scores across time in each patient. * indicates statistically significant difference between time 3 and time 1 in the pairwise comparison from TukeyHSD test following ANOVA. A P < 0.05 is considered statistically significant. Source data with the pairwise comparison of the p-values with Tukey HSD test are provided as a Source Data file.

Supplementary Figure 8: Key pathway contributors of the CDR archetype

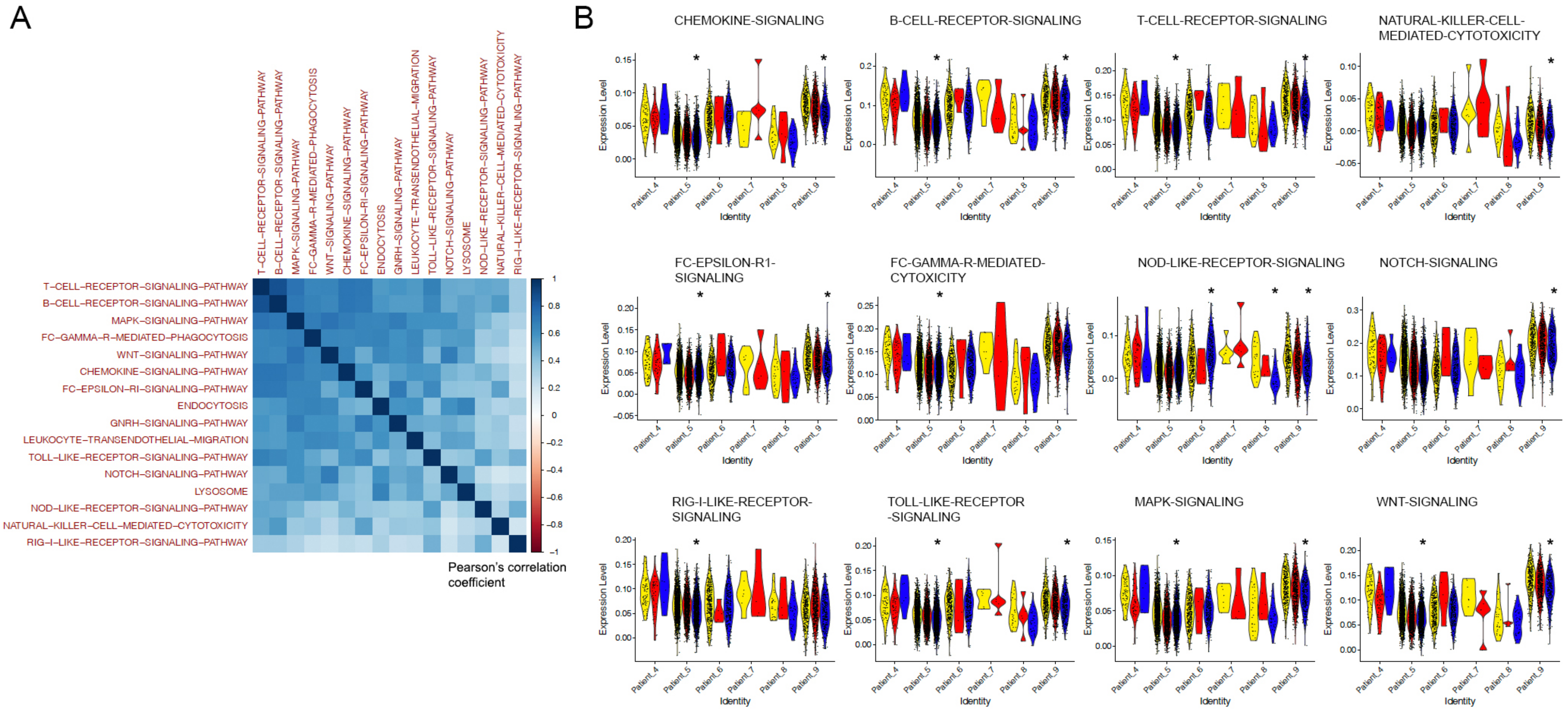

A. Correlation plot of key immune response related KEGG pathways across single cells classified as CDR specialists. The colors indicate magnitude of Pearson's correlation between the enrichment scores. B. Violin plots showing comparison of pathway enrichment scores across time in each patient. * indicates statistically significant difference between time 3 and time 1 in the pairwise comparison from Tukey HSD test (one-sided) following ANOVA. A $P < 0.05$ is considered statistically significant. Source data with the pairwise comparison of the p-values with Tukey HSD test are provided as a Source Data file.

Supplementary Figure 9: Expression levels of individual cell type identification markers in validation cohort

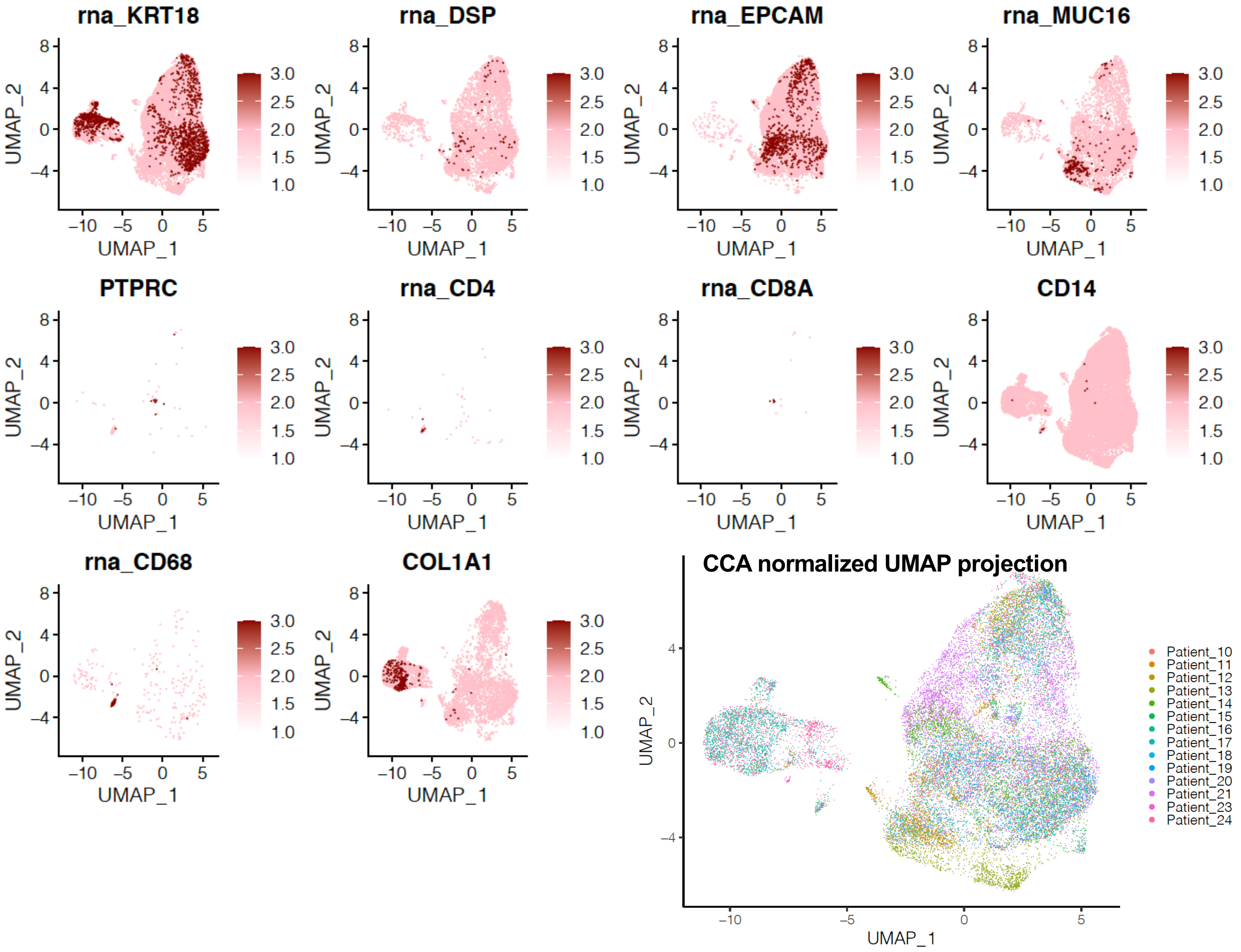

Supplementary Figure 10: Selecting optimal number of archetypes to enclose integrated validation cohort (Patients 10-24) scRNA-seq data

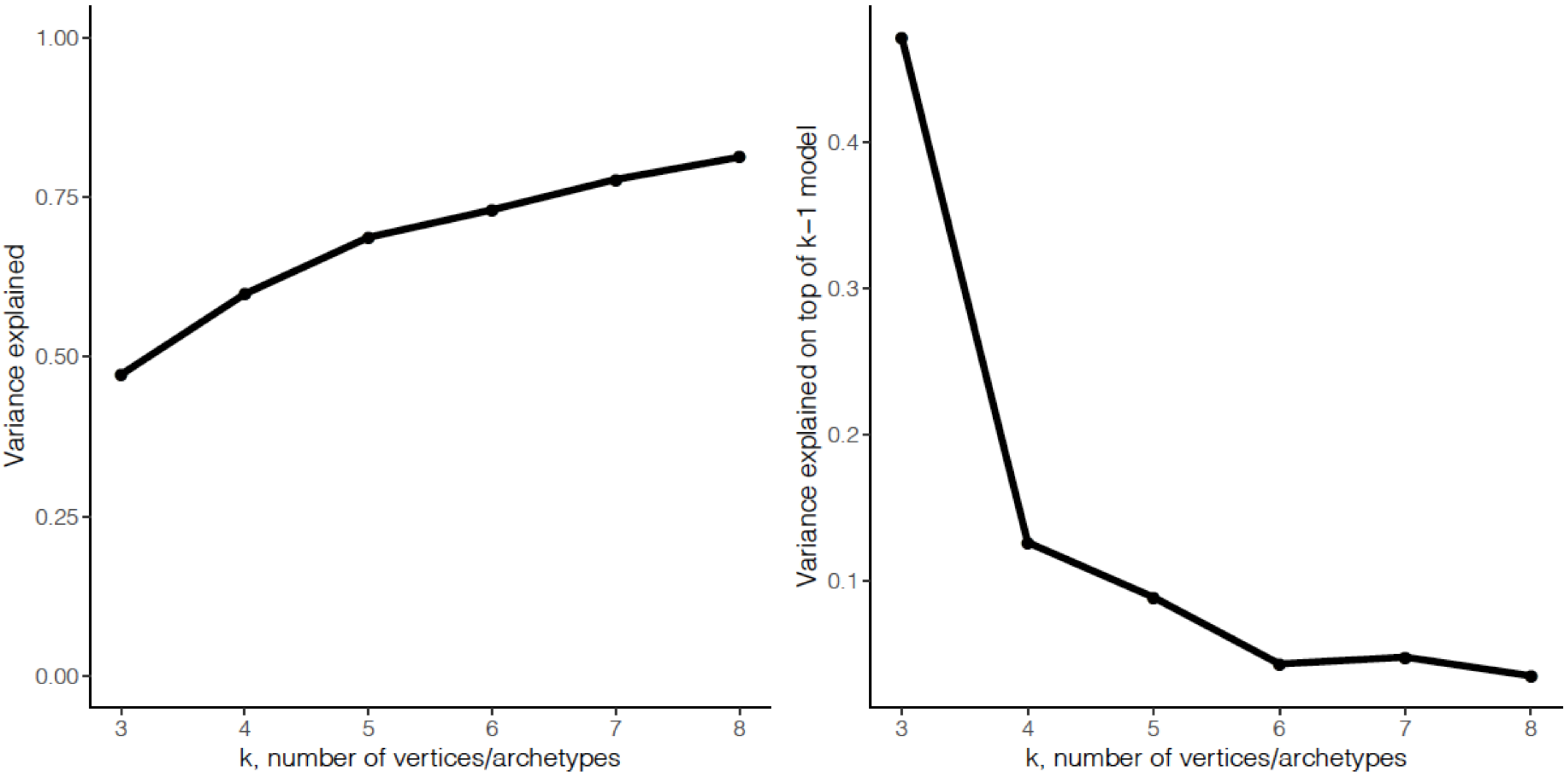

Variance explained by 3-8 archetypes (left panel), gain in variance explained in by each additional archetype (right panel)

Supplementary Figure 11: Pathway coefficients heatmap (validation cohort)

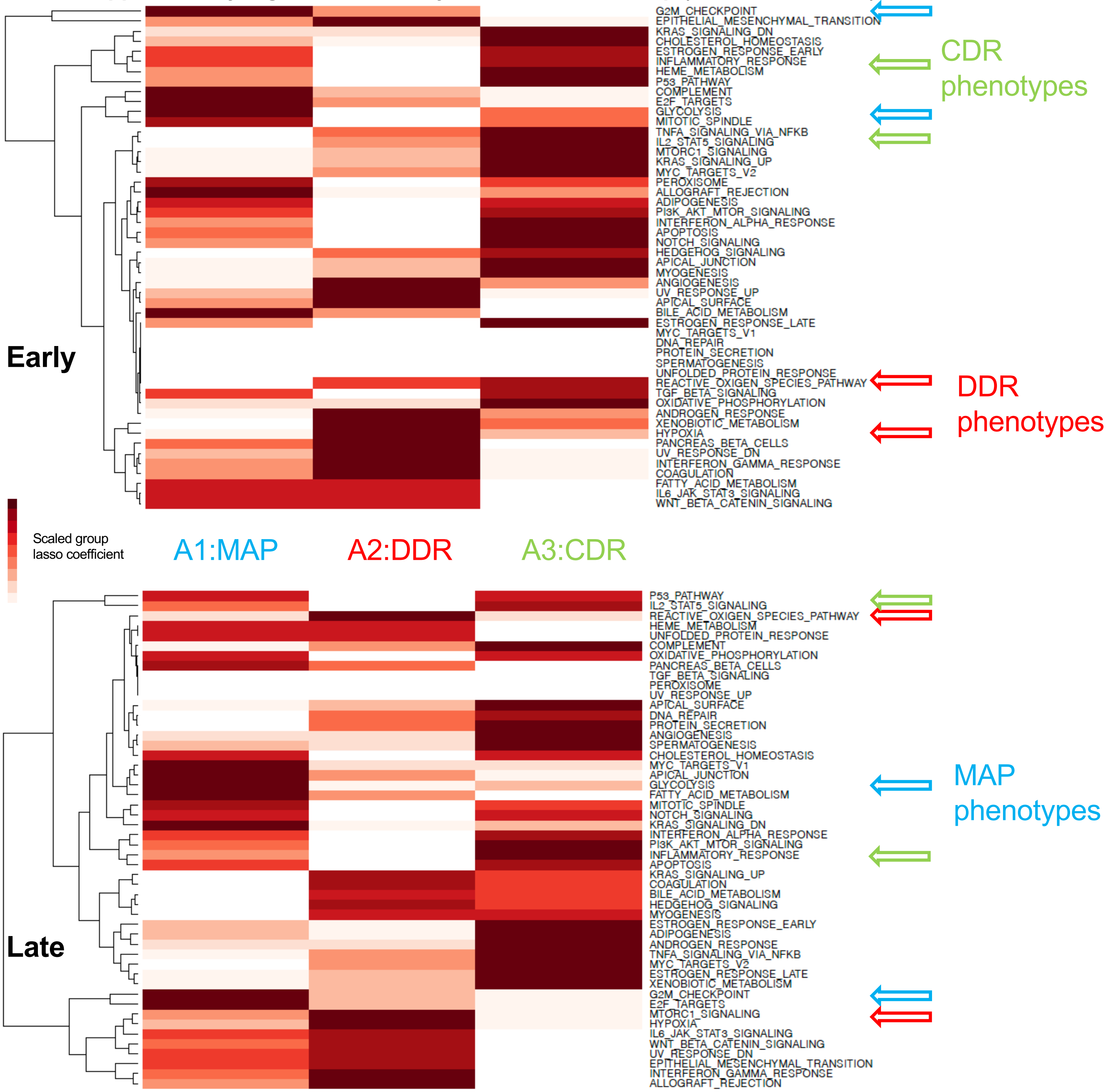

Supplementary Figure 12: Selecting optimal number of archetypes to enclose scRNA-seq data of individual longitudinal cohort patients

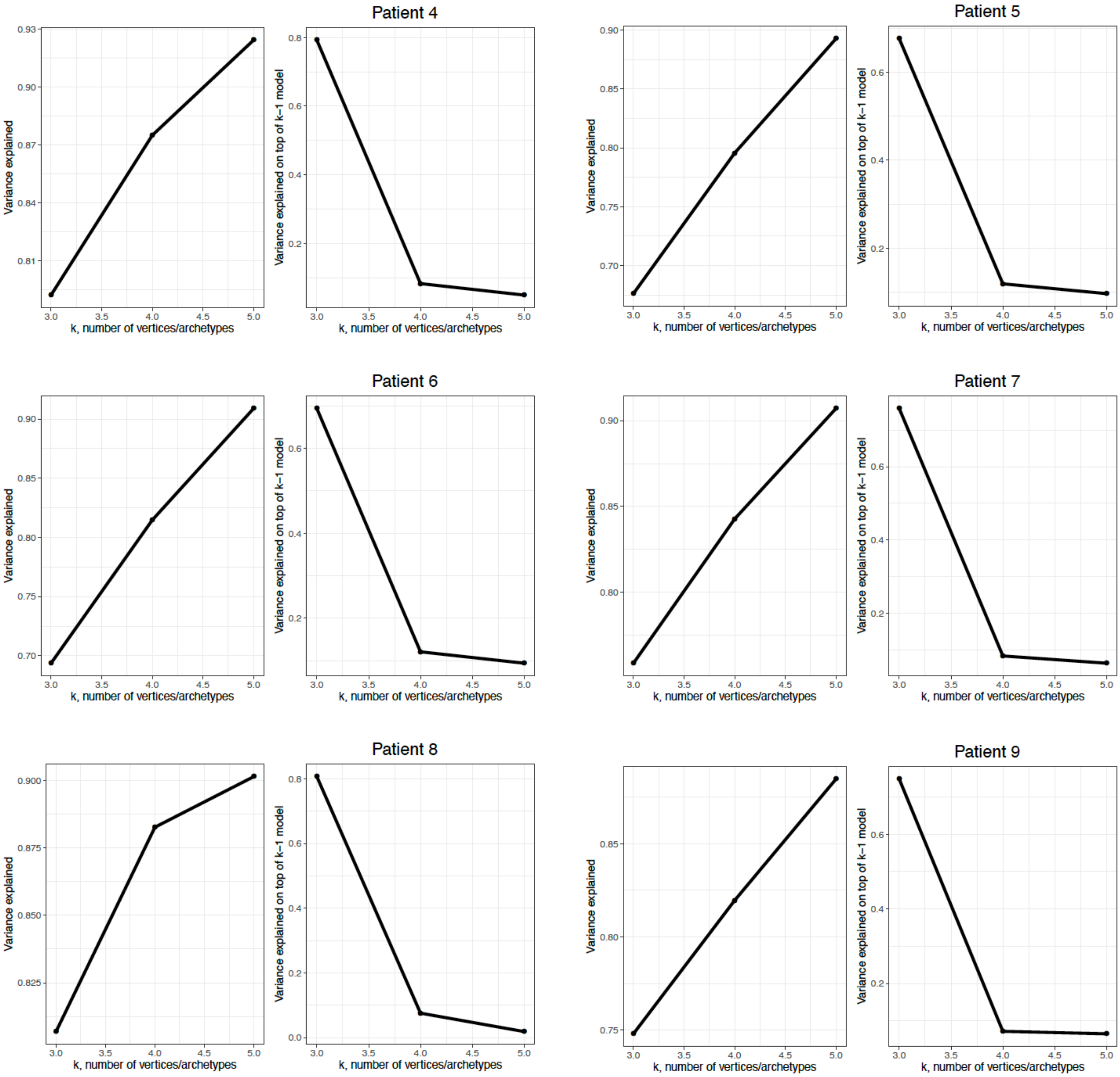

Supplementary Figure 13: Pathway coefficients and correlation heatmap (individual patients)

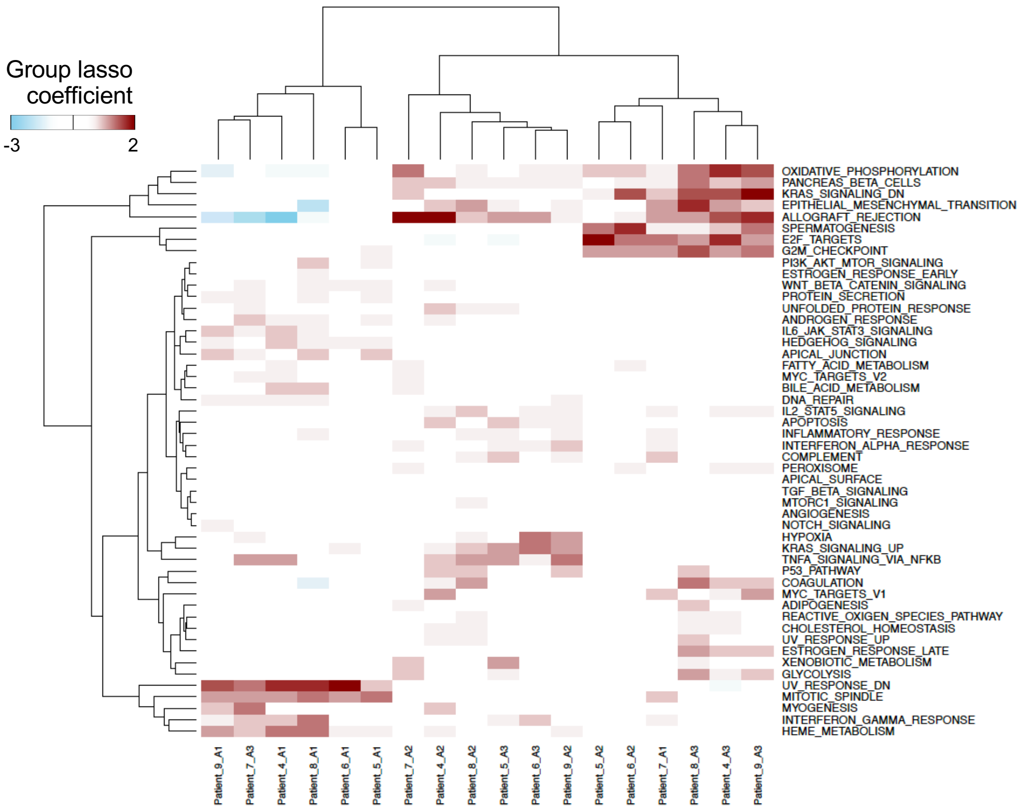

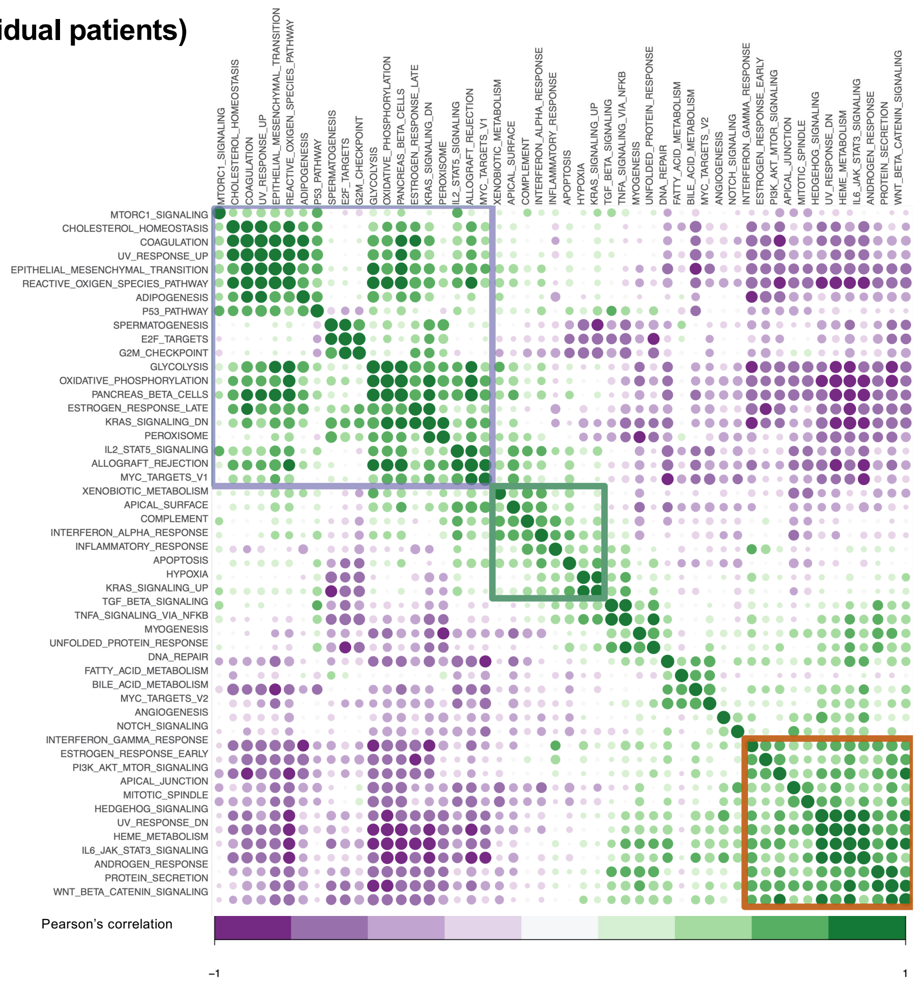

Supplementary Figure 14: Correlation between proportion of MAP specialists and CA-125 levels

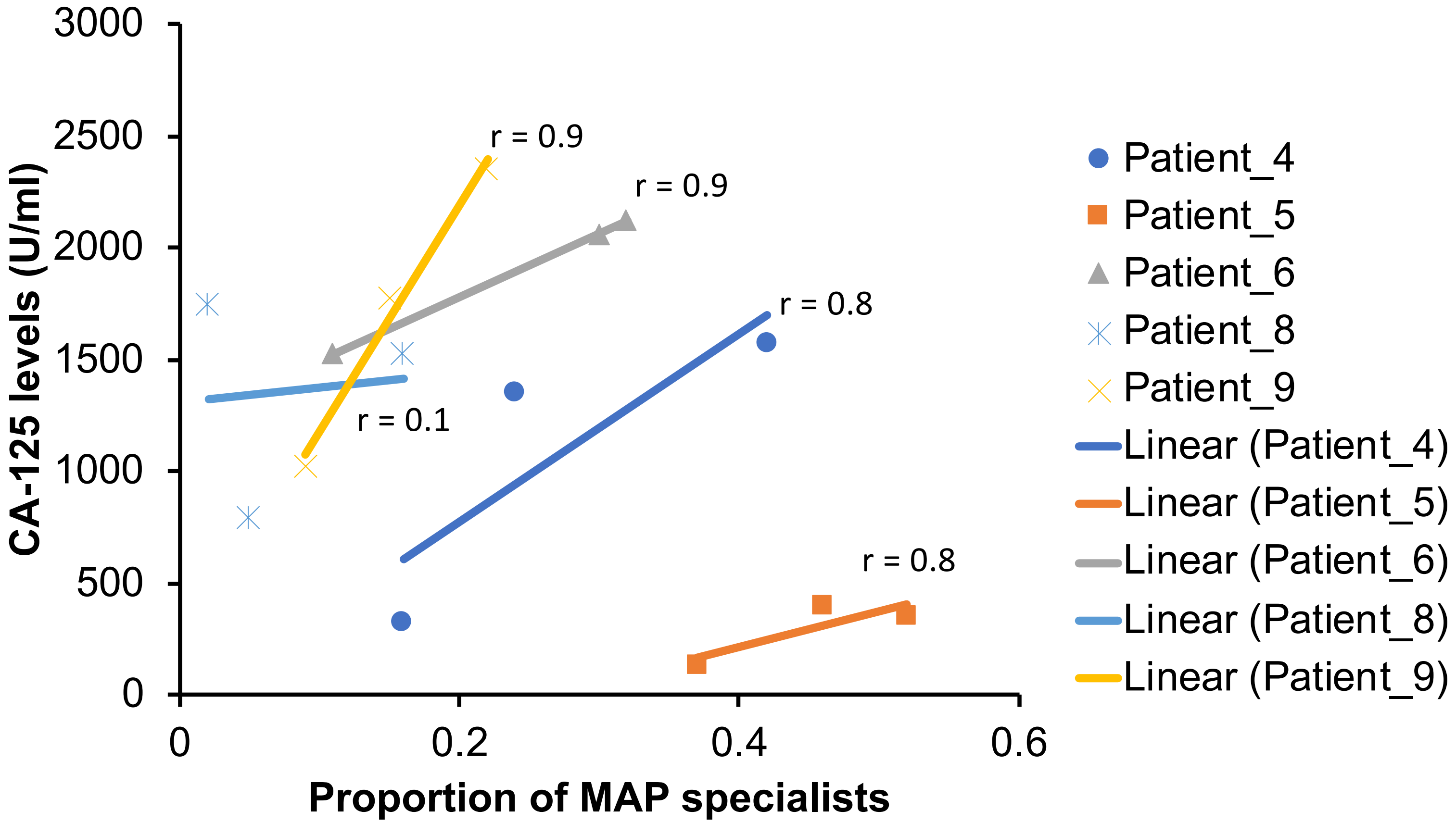

Scatterplot showing correlation between CA-125 levels and the proportion of MAP specialists in each patient. Pearson's correlation coefficient are displayed next to each linear fit. Data from Patient 7 is not displayed due to scRNA-seq data available only for two time points.

Supplementary Figure 15: Comparison of archetype specialists with transcriptional clusters, time and patient survival

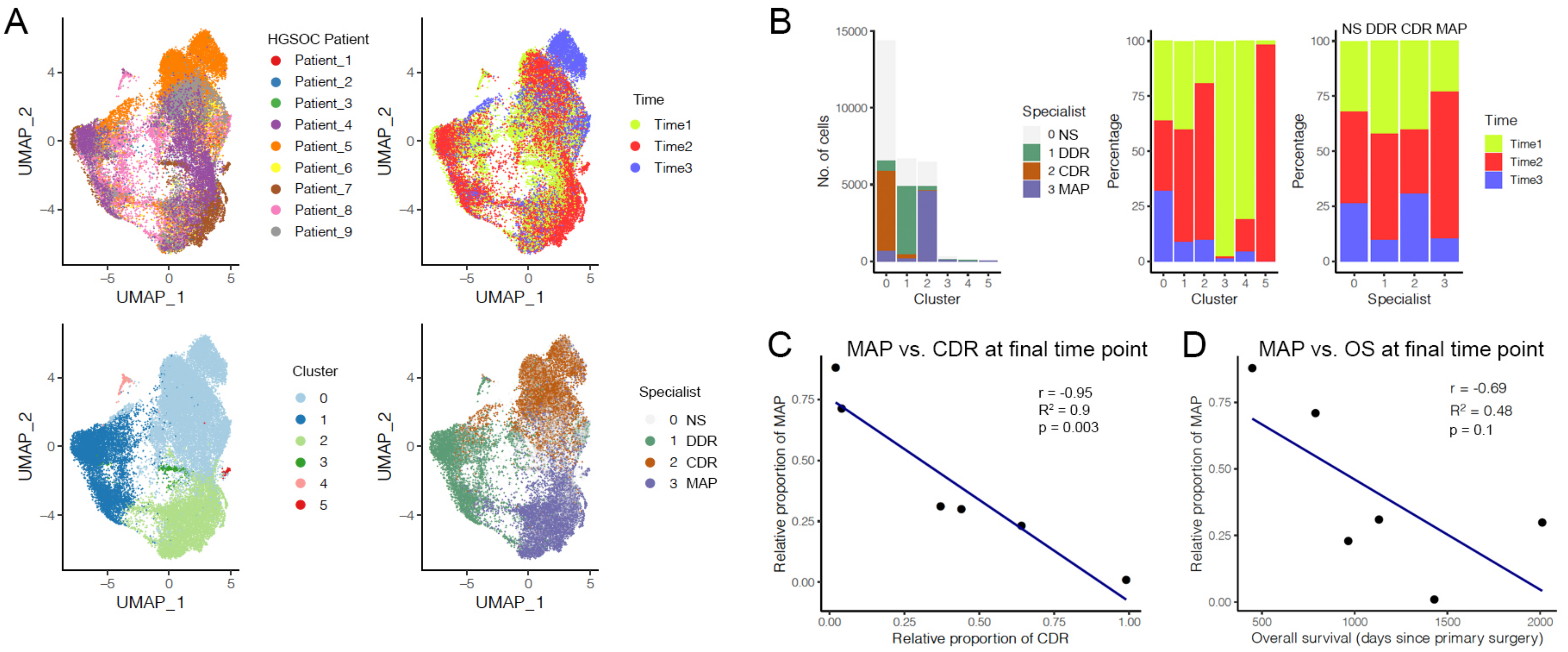

A. UMAP projections of batch-corrected cancer cells colored by patient (top left), time (top right), Seurat clusters (bottom left) or archetype specialist type (bottom right). B. Barplots showing absolute number of archetype specialists in different Seurat clusters (left panel), with stacked barplots (middle and right panels) showing percentage of cells in each Seurat cluster or specialist cluster grouped by time. C-D Scatterplots comparing the relative portion of MAP specialists at the last time point with the relative proportion of CDR specialists at the last time point (C) or overall survival in days since primary surgery was performed (D).

Supplementary Figure 16: Effect of fatty acid oxidation inhibition on total oxygen consumption rate across cell lines from patient samples collected at different time points

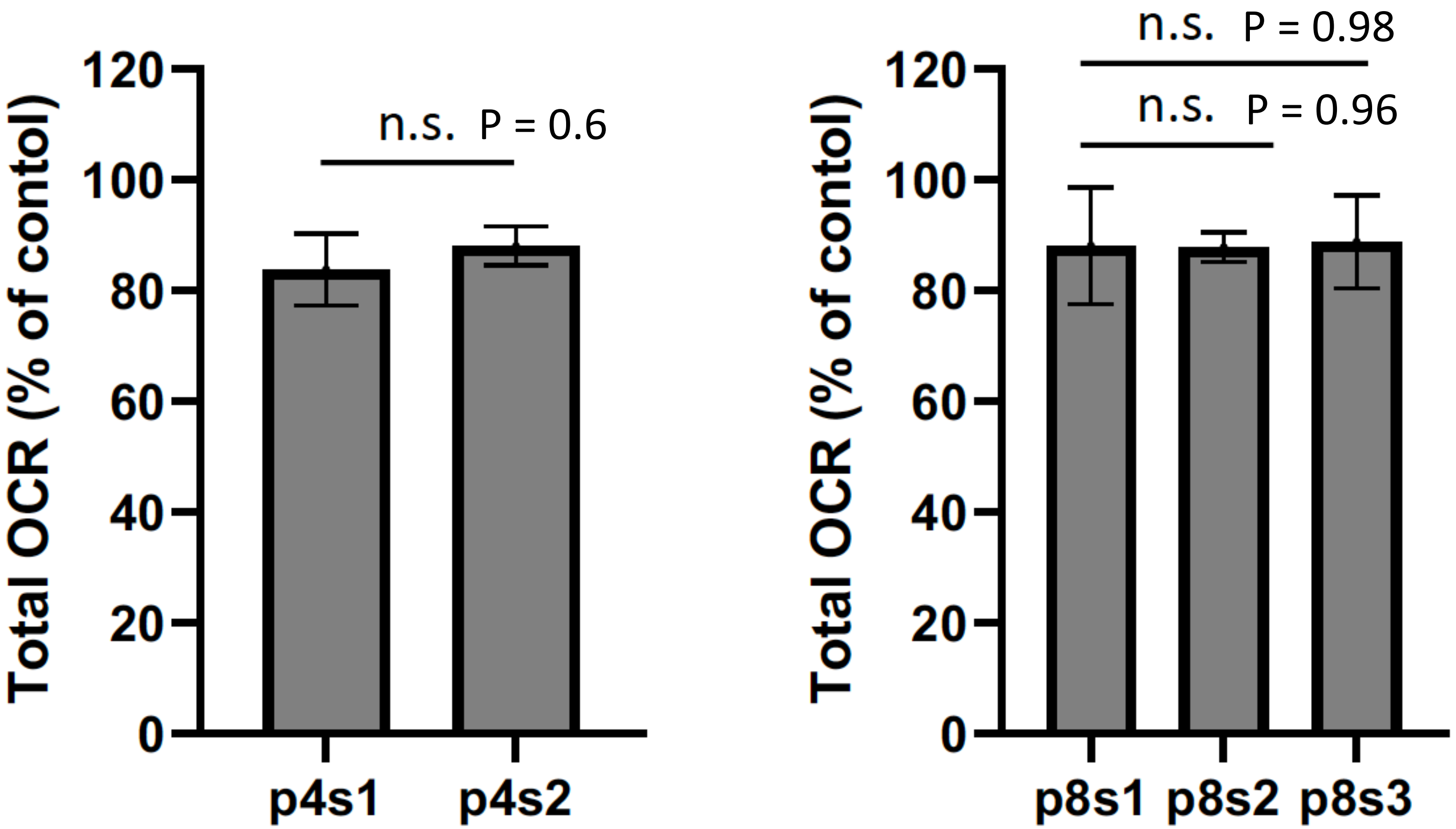

Barplots showing the mean and SEM (n = 3) of percentage total oxygen consumption rate in cell lines treated with fatty acid oxidation inhibitors. The left panel shows data from cell lines from patient 4 derived at the first and last time points, and the right panel shows data from cells lines from patient 8 at three time points. The horizontal bars above the plot show pairwise comparisons and results of two-tailed Student's t-test (n.s. = not significant).

Supplementary Figure 17: Distribution of archetype specialists based on presence of acquired somatic variants

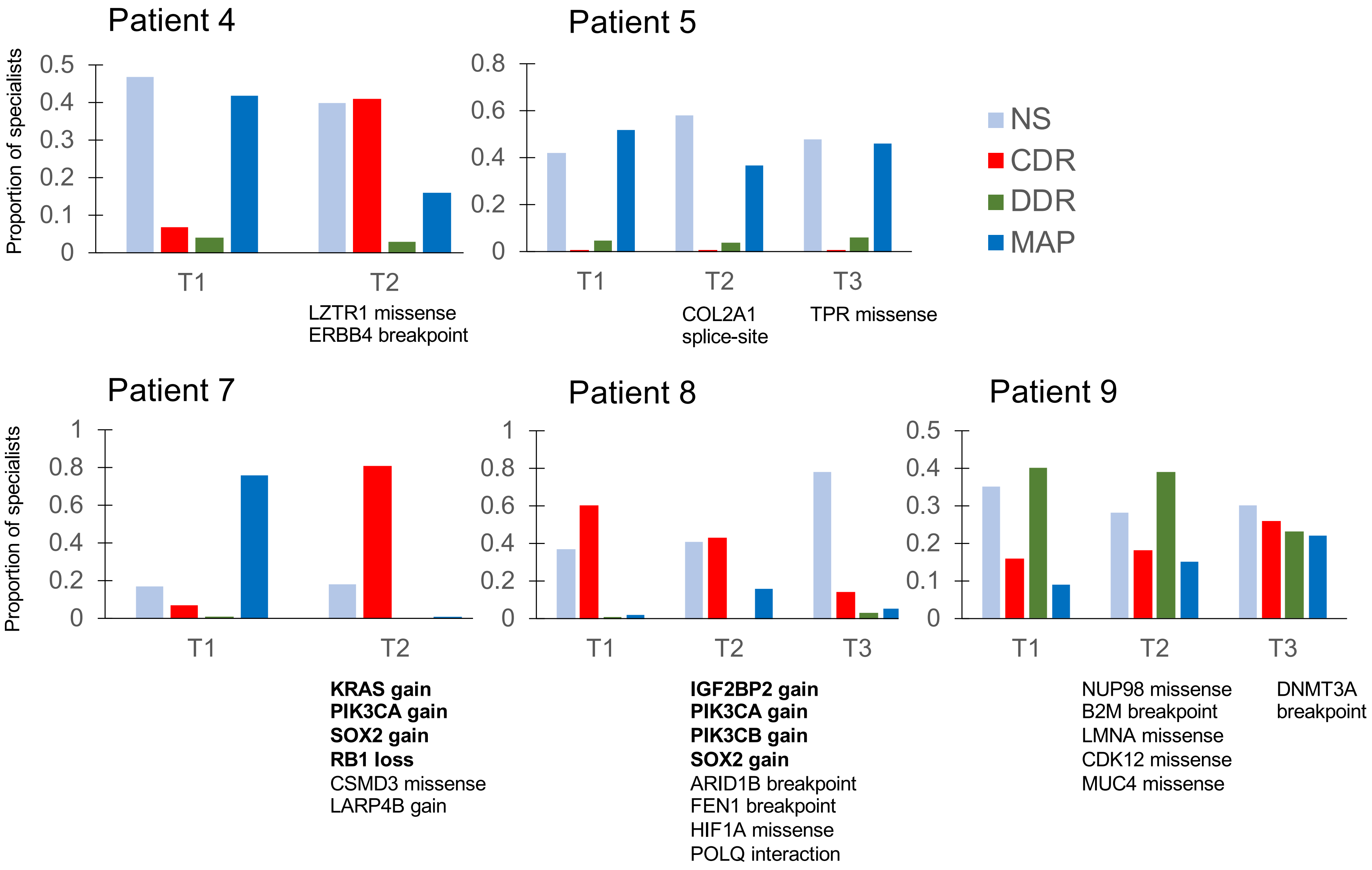

Barplots showing the proportion of each specialists across time points, and the somatic variants from WGS data that were acquired at the subsequent time points. The variants in bold are known pathogenic variants associated with HGSOC.

Supplementary Figure 18: Heatmaps of inferred CNV and estimated sub-clonal structure

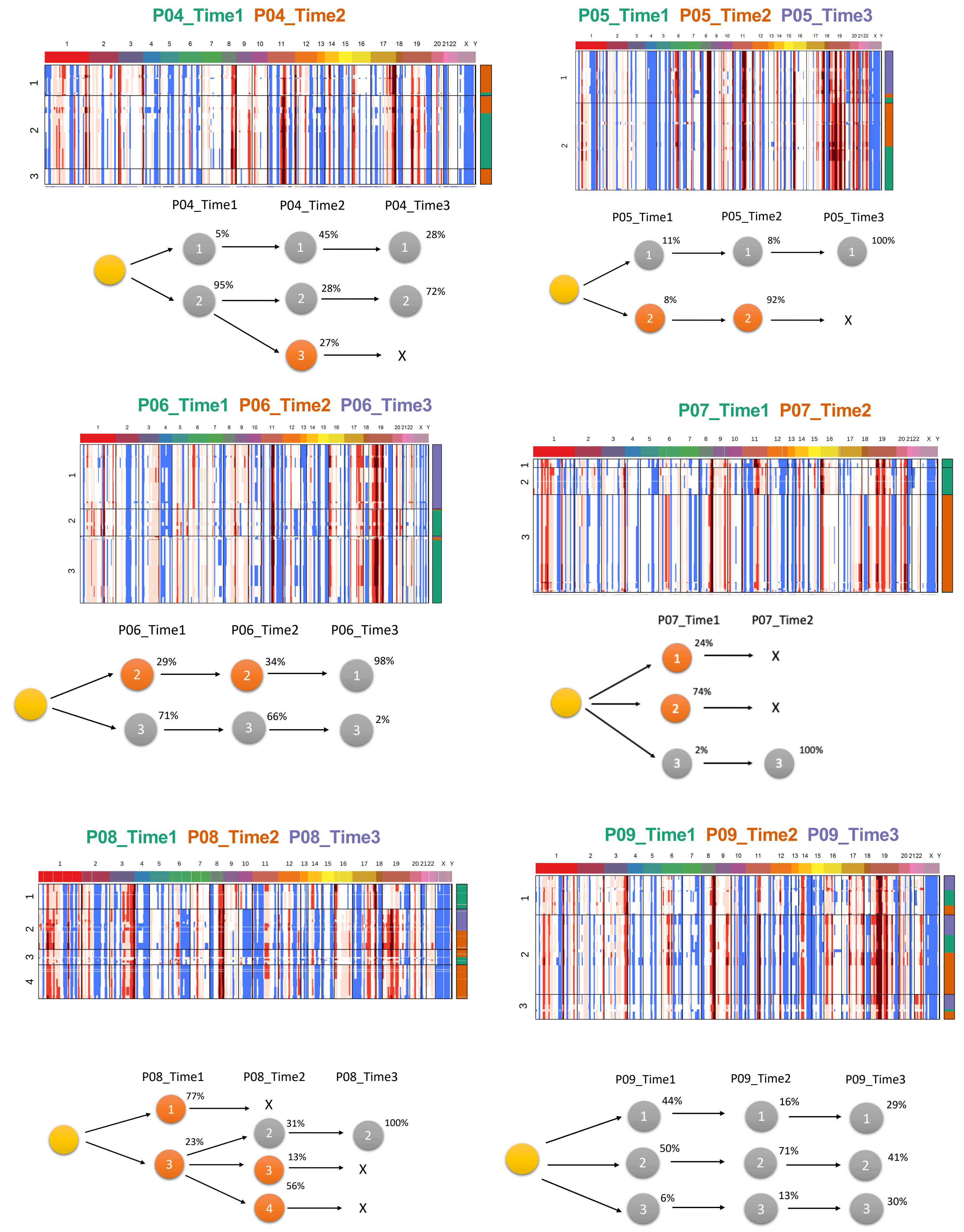

Supplementary Figure 19: Archetype score distribution by sub-clones determined from inferCNV

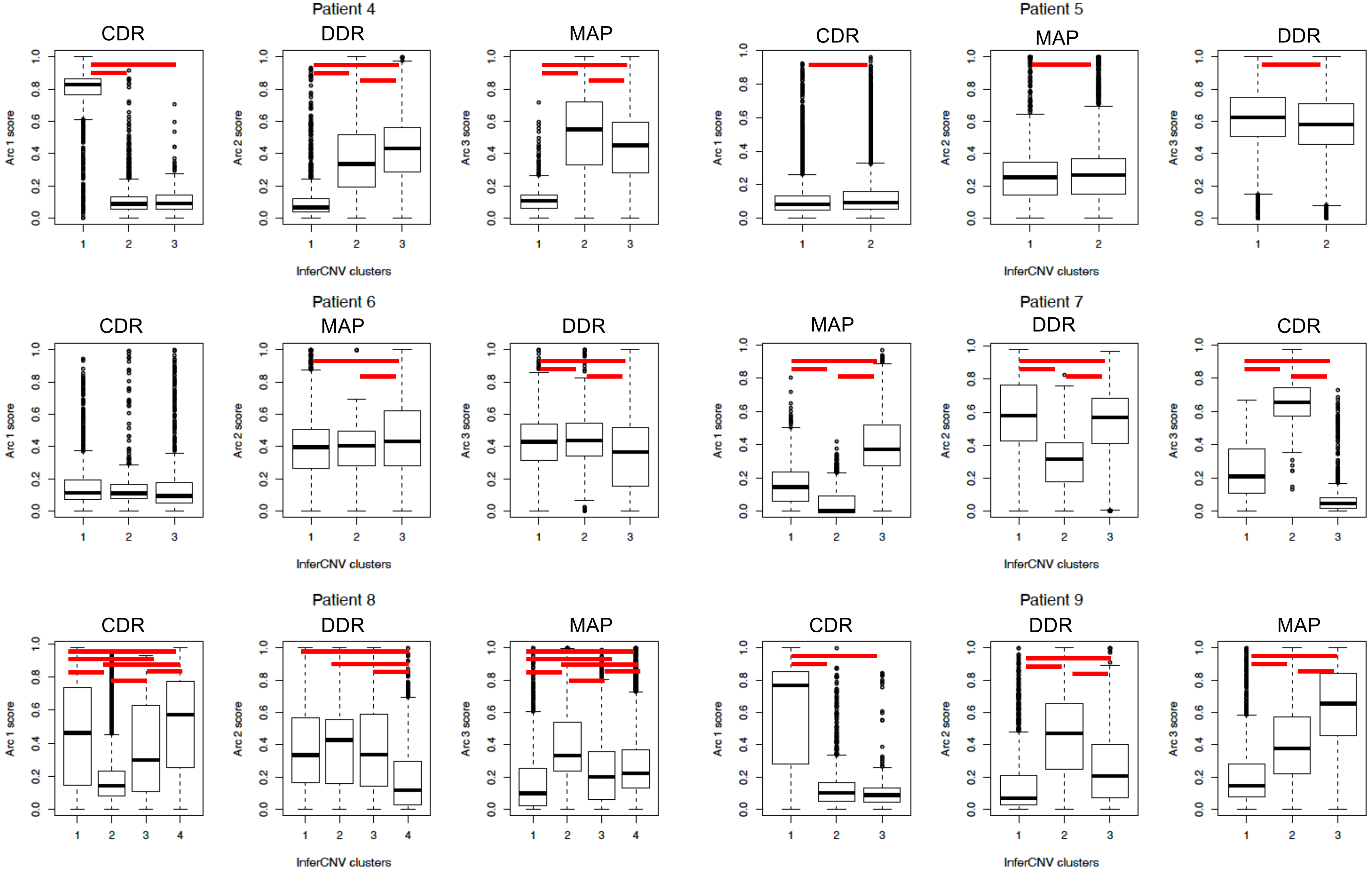

Indicates significant difference in mean archetype score between a pair of subclones (Tukey HSD adjusted P < 0.05 for pairwise comparisons)

Boxplots comparing archetype scores between InferCNV subclones in each patient. Significant pairwise p-values from Tukey's HSD posthoc analysis (single-sided) of the ANOVA model of the archetype scores against InferCNV subclone are shown in red. The lower and upper hinges in the boxplot indicate 25th and 75th percentiles, middle indicates 50th percentile (median), while the whiskers extended over 1.5 × interquartile range. Number of cells in each boxplot are listed below, followed by a complete list of pairwise comparisons from the Tukey HSD analysis. Source data with the pairwise comparison of the p-values with Tukey HSD test are provided as a Source Data file.

Patient	InferCNV subclone	N
P04	Subclone 1	926
P04	Subclone 2	2225
P04	Subclone 3	470
P05	Subclone 1	3008
P05	Subclone 2	5029
P06	Subclone 1	836
P06	Subclone 2	357
P06	Subclone 3	878
P07	Subclone 1	273
P07	Subclone 2	862
P07	Subclone 3	3119
P08	Subclone 1	1489
P08	Subclone 2	2407
P08	Subclone 3	917
P08	Subclone 4	2029
P09	Subclone 1	970
P09	Subclone 2	1972
P09	Subclone 3	618

Supplementary Methods

InferCNV analysis to confirm malignant epithelial cells and determining subclonal structure from scRNA-seq data

To identify malignant epithelial cells, we used a first-pass classification filter to separate epithelial cells from immune cells and stromal components. Since the samples were obtained from malignant pleural effusions and ascites, it was expected that the epithelial cells identified in the samples were malignant. These cells appeared in separate clusters in UMAPs and were verified with expression of key marker genes (Supplementary Figure 2). To further affirm the classification of the epithelial cells, we used the fibroblasts obtained from an HGSOC tumor as reference to perform inferCNV analysis with the epithelial cells from individual HGSOC patients across all time points. The parameters for the inferCNV analysis were: cutoff = 0.1 (as recommended for 10X scRNA-seq data), min_cells_per_gene = 3, with default hidden markov model (HMM) and denoise set to true. The resulting profiles displayed the presence of CNAs in all epithelial cells compared to the normal reference, thus confirming the classification of malignant epithelial cells. The heatmaps on the subsequent pages also display the expression of key epithelial markers, such as *EPCAM*, *KRT8* and *KRT18* across the single cells, along with the absence of immune cell markers like *CD45, THY1, CD3E* and *CD68*.

To construct the subclonal structure for each patient, we used the subcluster method with the HMM CNA profiles to first determine the number of subclones and distribution of the cells in each subclone. Next, we inspected the HMM and denoised CNA profiles

to identify regions of copy number gains or losses that could verify the subclonal structure determined by the HMM subcluster method. We also used the whole genome sequencing profiles in case of patients where the regions of gain or losses that distinguished subclones were difficult to assign using the HMM or CNA profiles alone. After the subclonal structures were confirmed and individual cells assigned to each subclone, we derived the evolutionary tree as follows: First, the proportion of cells assigned to each subclone were used to determine the frequency of subclones at each time point. Then, the branches of the tree were assigned on the basis of shared regions of gain or loss.

For example, in the case of Patient 4 (see figure on next page) the three subclones were first determined using the HMM subclusters. Then, Subclone 1 was distinguished from subclones 2 and 3 by the presence of a unique region of gain on chromosomes 1 and 17. Subclones 2 and 3 shared a difference in region of gain on chromosome 17 seen in subclone 2, while subclone 3 was characterized by unique regions of loss on chromosomes 4 and X. These regions are encircled in black. Thus, we determined that subclone 3, which appears in samples collected at the second time point, evolved from subclone 2. Finally, the distribution of the cells in the three subclones were used to define the subclone frequencies at each time point. This procedure was followed for other samples, where the HMM subclusters were first confirmed by the presence of unique regions of gain or loss, followed by assignment of the evolutionary branching based on shared and acquired CNAs, and determination of subclone frequencies.

The figure panels below show the inferred CNAs in the malignant epithelial cells of each patient sample along with the reference cells. The heatmaps below indicate the regions of gain or loss (encircled in black) that were used to confirm the HMM subclusters and assign the evolutionary branches.

PATIENT_4

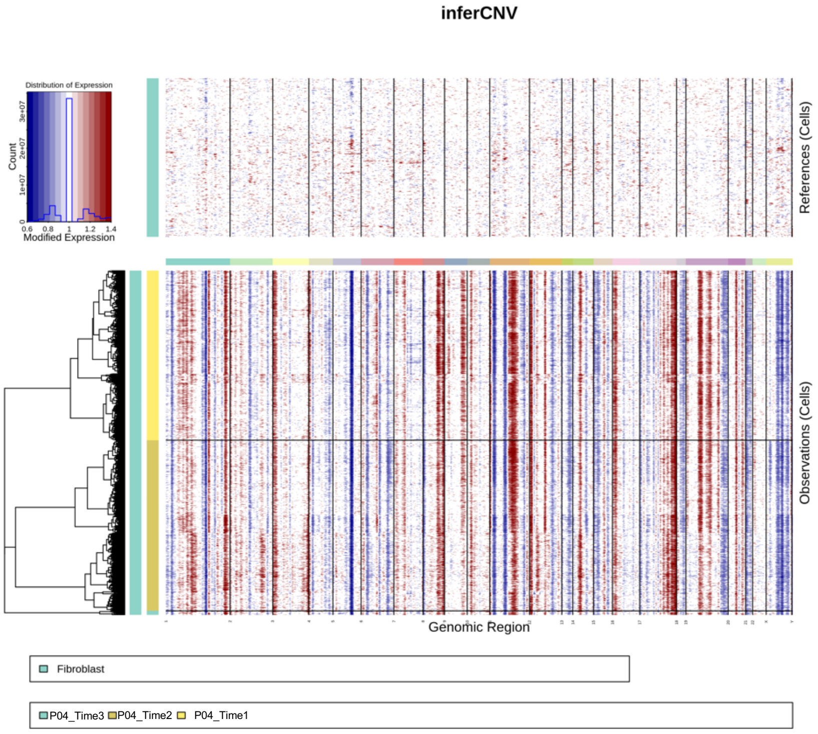

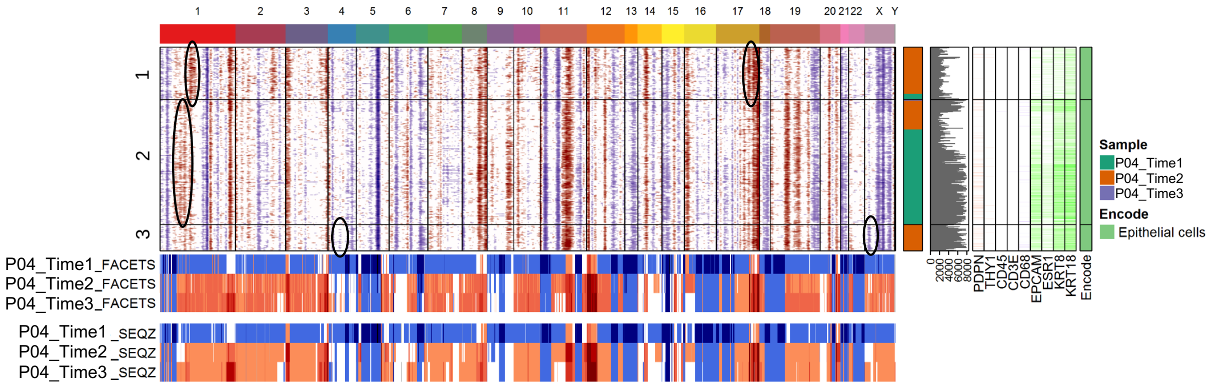

Patient_4 subclones: Subclone 1 determined by gains on Chr 1 and 17, subclone 2 determined by gain on chr 1, subclone 3 branch from subclone determined by shared region of no gain on chr 17, and unique loss on chr 4 and X.

PATIENT_5

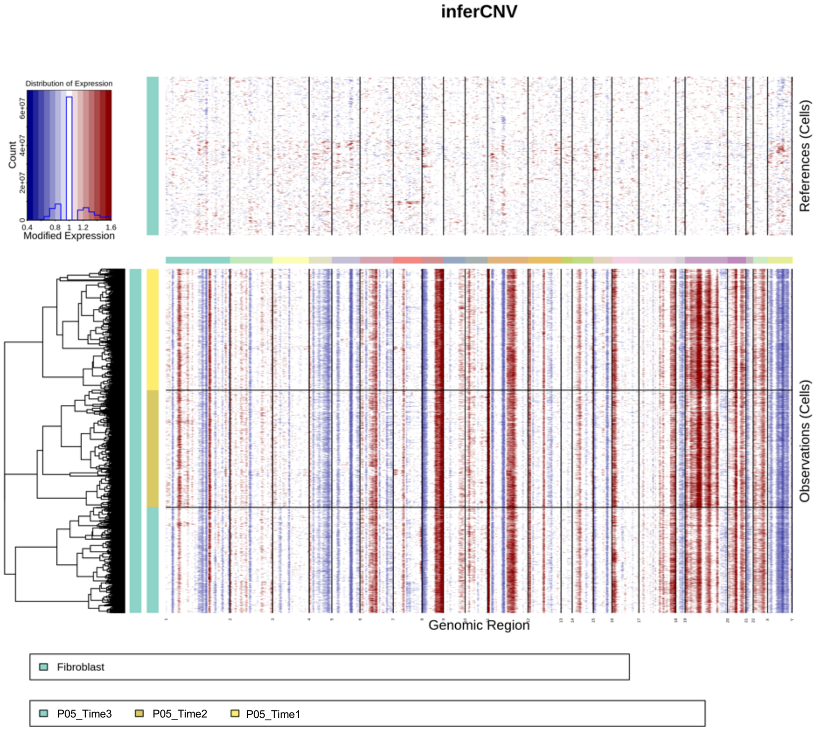

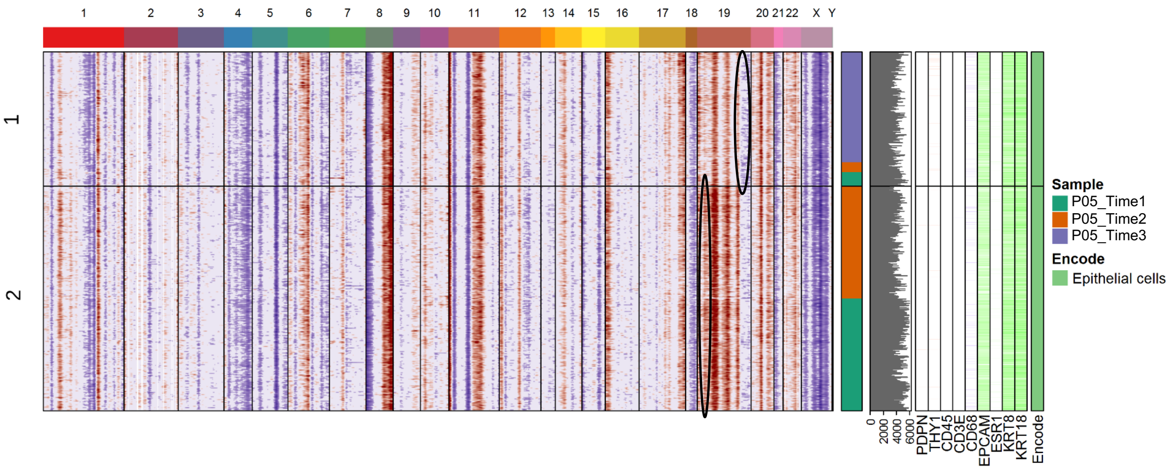

Patient_5 subclones: Subclone 2 distinguished from subclone 1 by gain on chr 19.

PATIENT_6

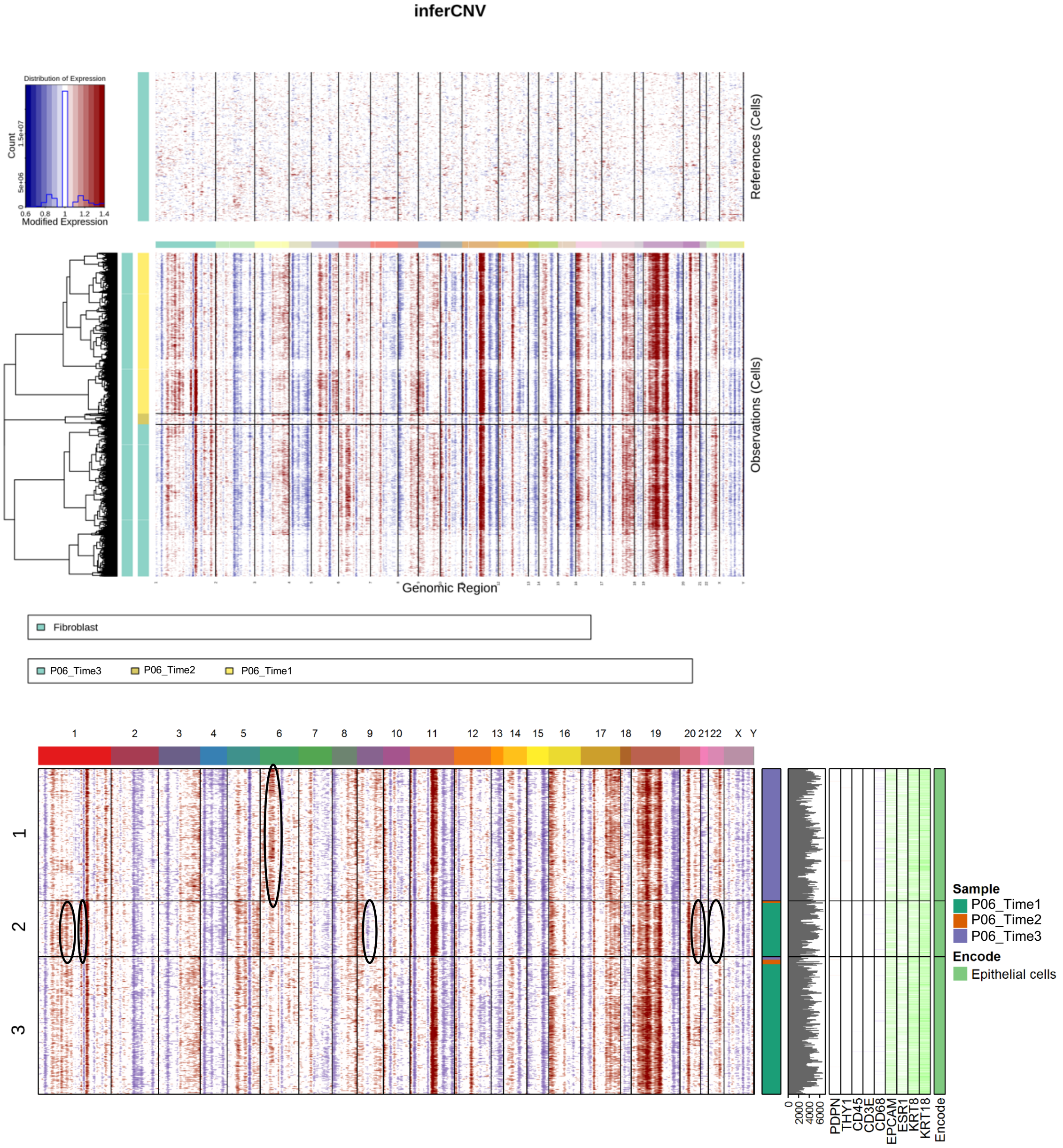

Patient_6 subclones: Subclone 1 determined by gains on chr 6, subclone 2 determined by gain on chr 1, loss on chr 9, gain on chr21, subclone 3 did not share regions of gain or loss with subclones 1 and 2. Subclone 1 branch from subclone 3 determined based on shared CNA pattern besides gain on chr 6 acquired in subclone 1.

PATIENT_7

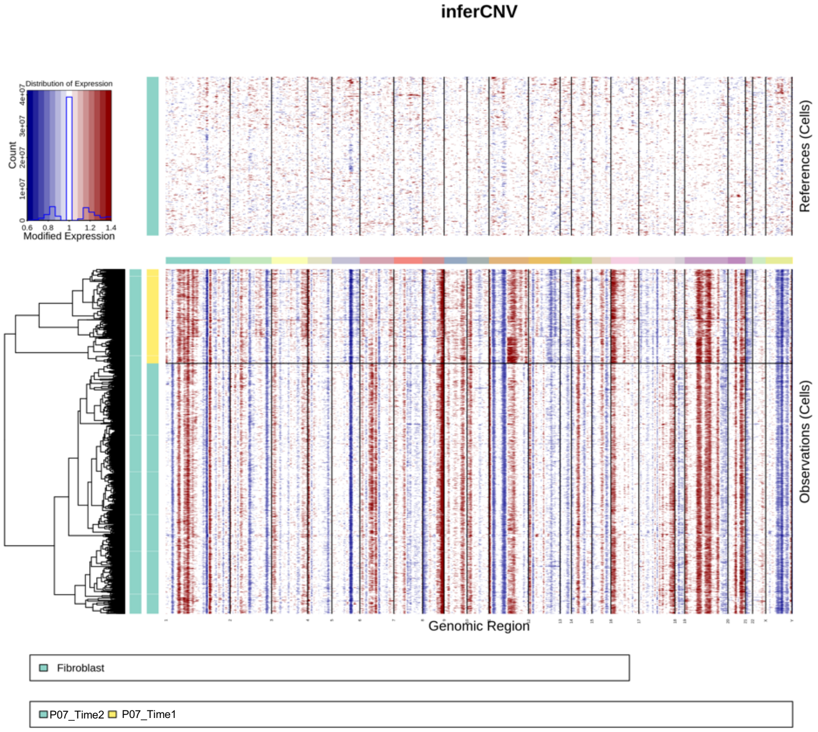

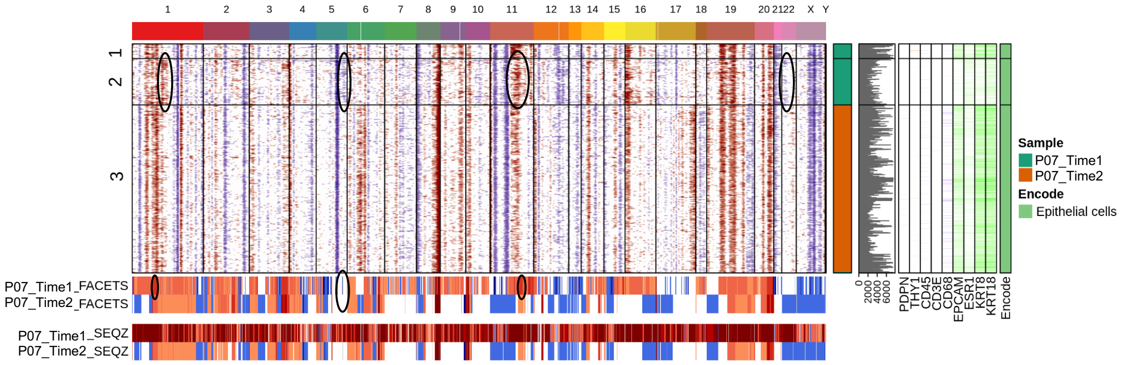

Patient_7 subclones: Subclone 1 determined by gain on chr 11, subclone 2 determined by gains on chr 1 and 11, loss on chr 22, subclone 2 determined by gain on chr 1. Subclone 3 did not share CNA patterns with subclones 1 and 2.

PATIENT_8

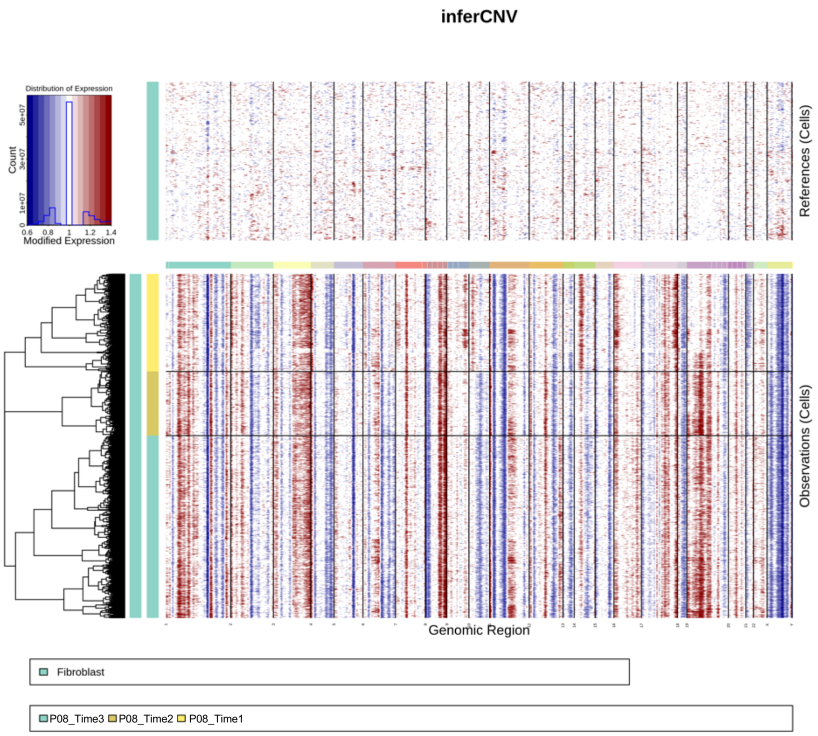

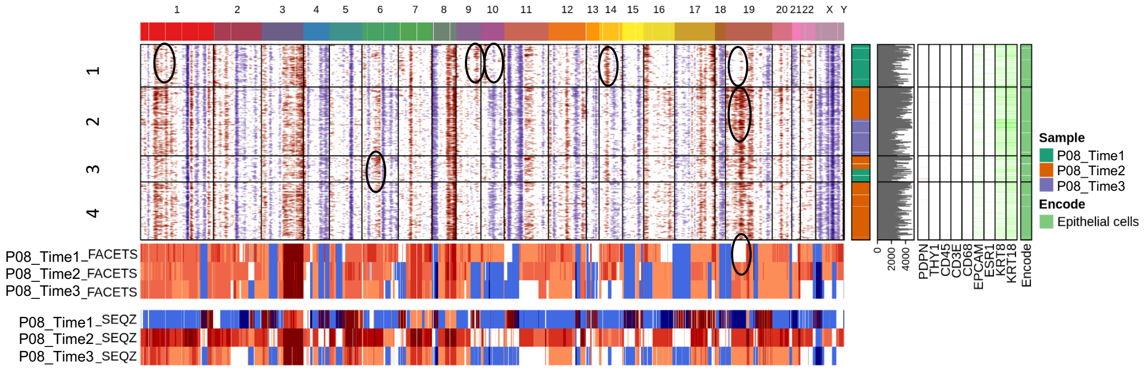

Patient_8 subclones: Subclone 1 determined by gains on chr 1, 9 and 14 and no gain on chr 19, subclone 2 (surviving subclone at last time point) determined by additional gain on chr 19 from subclone 3, subclone 4 distinguished from subclone 3 based on difference in region of gain on chr 6.

PATIENT_9

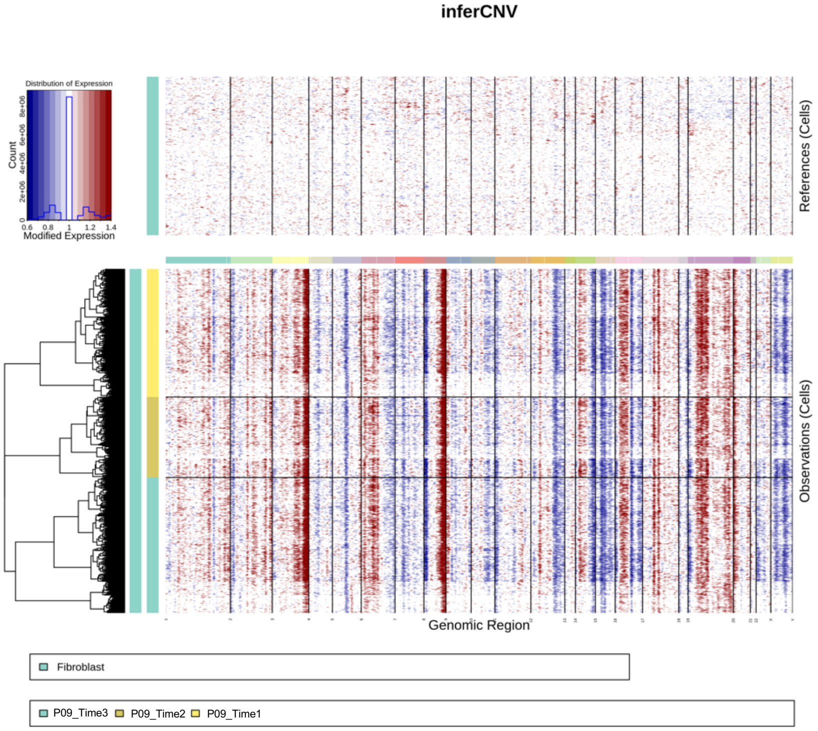

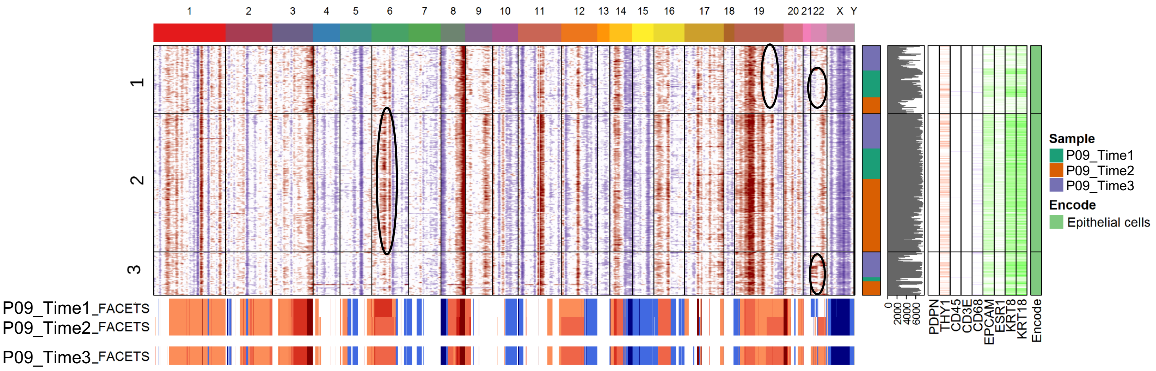

Patient_9 subclones: Subclone 1 determined by region of no gain on chr 19 and loss on chr 22, subclone 2 determined by gain on chr 6, subclone 3 based on gain in chr 22.
