# Supplementary material for: Evolution of core archetypal phenotypes in progressive high grade serous ovarian cancer
Source: Nat Commun. 2021 May 24;12:3039. doi: 10.1038/s41467-021-23171-3 (PMC8144406; doi:10.1038/s41467-021-23171-3)
Supplement: Supplementary file 3 — Description of Additional Supplementary Files [file 41467_2021_23171_MOESM3_ESM.pdf]

Description of Additional Supplementary Files

File Name: Supplementary Data 1
Description: Longitudinal cohort overall survival

File Name: Supplementary Data 2
Description: Longitudinal cohort treatment history

File Name: Supplementary Data 3
Description: Longitudinal cohort CA-125 levels

File Name: Supplementary Data 4
Description: Validation cohort treatment history

File Name: Supplementary Data 5
Description: Linear regression analysis of KEGG pathways against archetype scores

File Name: Supplementary Data 6
Description: Proportions of archetype specialists

File Name: Supplementary Data 7
Description: Summary of WGS analysis

File Name: Supplementary Data 8
Description: WGS Germline Variants

File Name: Supplementary Data 9
Description: High impact somatic variants

File Name: Supplementary Data 10
Description: WGS copy number estimates (z-transformed)

File Name: Supplementary Data 11
Description: WGS genic structural variants
