# Supplementary material for: Evolution of core archetypal phenotypes in progressive high grade serous ovarian cancer
Source: Nat Commun. 2021 May 24;12:3039. doi: 10.1038/s41467-021-23171-3 (PMC8144406; doi:10.1038/s41467-021-23171-3)
Supplement: Supplementary file 5 — Reporting Summary [file 41467_2021_23171_MOESM5_ESM.pdf]

nature research

Corresponding author(s): Bowtell and Bild

Last updated by author(s): Mar 17, 2021

Reporting Summary

Nature Research wishes to improve the reproducibility of the work that we publish. This form provides structure for consistency and transparency in reporting. For further information on Nature Research policies, see our Editorial Policies and the Editorial Policy Checklist.

Statistics

For all statistical analyses, confirm that the following items are present in the figure legend, table legend, main text, or Methods section.

n/a	Confirmed	
☐	☒	The exact sample size (n) for each experimental group/condition, given as a discrete number and unit of measurement
☐	☒	A statement on whether measurements were taken from distinct samples or whether the same sample was measured repeatedly
☐	☒	The statistical test(s) used AND whether they are one- or two-sided<br>*Only common tests should be described solely by name; describe more complex techniques in the Methods section.*
☐	☒	A description of all covariates tested
☐	☒	A description of any assumptions or corrections, such as tests of normality and adjustment for multiple comparisons
☐	☒	A full description of the statistical parameters including central tendency (e.g. means) or other basic estimates (e.g. regression coefficient) AND variation (e.g. standard deviation) or associated estimates of uncertainty (e.g. confidence intervals)
☐	☒	For null hypothesis testing, the test statistic (e.g. F, t, r) with confidence intervals, effect sizes, degrees of freedom and P value noted<br>*Give P values as exact values whenever suitable.*
☒	☐	For Bayesian analysis, information on the choice of priors and Markov chain Monte Carlo settings
☒	☐	For hierarchical and complex designs, identification of the appropriate level for tests and full reporting of outcomes
☐	☒	Estimates of effect sizes (e.g. Cohen's d, Pearson's r), indicating how they were calculated

Our web collection on statistics for biologists contains articles on many of the points above.

Software and code

Policy information about availability of computer code

Data collection	No software was used for data collection.
Data analysis	Open source software: R-Studio (1.2.5033) with R (3.6.3); Seurat (3.1), GSVA (1.34.0), glmnet (4.0-2), SingleR (1.0.6), InferCNV (1.2.1), RCircos (1.2.1), ParetoTI (https://github.com/vitkl/ParetoTI);<br>ParTI package for MATLAB (https://github.com/AlonLabWIS/ParTI);<br>Code for archetype analyses and multitask learning : https://github.com/U54Bioinformatics/03A_scRNA_Archetype_Mutitasklearning_Analysis<br>Standalone open source software that were used in the BETSY pipeline (https://github.com/jefftc/changlab): Cell Ranger (>2.1.1), STAR (>2.6), featureCounts (2.0), BWA-MEM (0.7.17), Sequenza (3.0), FACETS (0.5), strelka (2.9), Mutect2, Muse, SVaba (1.1).<br>Custom pipelines for BETSY: https://github.com/U54Bioinformatics/01B_Preprocess_DNAseq, https://github.com/U54Bioinformatics/01A_Preprocess_scRNAseq, https://github.com/U54Bioinformatics/02C_scRNAseq_Pathway ;<br>Commercial software: BIOTEK GEN5 (3.0.5), GraphPad (Prism 8.4.3), MATLAB (R2020b), Agilent WAVE (2.6.1), ICELL8 CellSelect (1.1.10.0)

For manuscripts utilizing custom algorithms or software that are central to the research but not yet described in published literature, software must be made available to editors and reviewers. We strongly encourage code deposition in a community repository (e.g. GitHub). See the Nature Research guidelines for submitting code & software for further information.

Data

Policy information about availability of data

All manuscripts must include a data availability statement. This statement should provide the following information, where applicable:

- Accession codes, unique identifiers, or web links for publicly available datasets
- A list of figures that have associated raw data
- A description of any restrictions on data availability

Single-cell RNA-Seq data generated and analyzed during this study are available from the GEO database under accession GSE158722 (https://www.ncbi.nlm.nih.gov/geo/query/acc.cgi?acc=GSE158722).

Whole-genome sequencing and raw scRNA-seq data are available under controlled access from dbGaP under the accession ID phs002294 (https://www.ncbi.nlm.nih.gov/projects/gap/cgi-bin/study.cgi?study_id=phs002294.v1.p1).

Catalog of driver genes from IntOGen are publicly available (https://www.intogen.org/download?file=IntOGen-Drivers-20200201.zip).

Source raw data associated with the ATP assays (Figure 4c-d) and p-values from multiple comparisons analyzed using Tukey HSD test (Supplementary Figures 7c-d, Supplementary Figure 8b and Supplementary Figure 19) are provided with this paper as Source Data file.

Field-specific reporting

Please select the one below that is the best fit for your research. If you are not sure, read the appropriate sections before making your selection.

- [x] Life sciences
- [ ] Behavioural & social sciences
- [ ] Ecological, evolutionary & environmental sciences

For a reference copy of the document with all sections, see nature.com/documents/nr-reporting-summary-flat.pdf

Life sciences study design

All studies must disclose on these points even when the disclosure is negative.

Sample size	No variables were predetermined prior to this study for measurement and power calculations. This was a retrospective study of individual patient single-cell transcriptomes and genomes. All patients collected through our protocol with viable samples were used. Final sample size was determined from available cohort of collected patient samples in which cancer cell isolation yield was sufficient to perform scRNA-seq.
Data exclusions	Low quality single cells were excluded from further analysis, defined as cells with <1000 genes, >25% mitochondrial reads or counts <2000 or >80,000.
Replication	An independent validation cohort of 14 samples were sequenced and confirmed to verify results from initial cohort of 9 patient samples. For in vitro experiments, data from three independent biological replicates were obtained.
Randomization	Not Applicable - retrospective scRNA-seq and WGS study of all available previously collected patient samples.
Blinding	Not applicable - retrospective scRNA-seq and WGS study of all available previously collected patient samples.

Reporting for specific materials, systems and methods

We require information from authors about some types of materials, experimental systems and methods used in many studies. Here, indicate whether each material, system or method listed is relevant to your study. If you are not sure if a list item applies to your research, read the appropriate section before selecting a response.

Materials & experimental systems

n/a	Involved in the study	
[ ]	[x]	Antibodies
[x]	[ ]	Eukaryotic cell lines
[x]	[ ]	Palaeontology and archaeology
[x]	[ ]	Animals and other organisms
[ ]	[x]	Human research participants
[x]	[ ]	Clinical data
[x]	[ ]	Dual use research of concern

Methods

n/a	Involved in the study	
[x]	[ ]	ChIP-seq
[x]	[ ]	Flow cytometry
[x]	[ ]	MRI-based neuroimaging

Antibodies

Antibodies used	biotinylated anti-podoplanin antibody, Biolegend, Cat# 337015
Validation	The antibody was purified by affinity chromatography, and conjugated with biotin<br>under optimal conditions. Each lot of this antibody is quality control tested by immunofluorescent staining with flow cytometric analysis. https://www.biolegend.com/en-us/products/biotin-anti-human-podoplanin-antibody-9661?GroupID=GROUP28

Human research participants

Policy information about studies involving human research participants

Population characteristics	Ovarian cancer (all female), 44 to 67 years of age at diagnosis, high-grade, serous, treatment-naive or 3 to 11 lines of treatment.
Recruitment	N/A (leftover samples were used in this study).
Ethics oversight	Samples were collected under IRB # 07047 & 17334 (City of Hope), 41030 and 89989 (University of Utah), or HREC # 01/60, 16/161 by the Australian Ovarian Cancer Study (AOCS) which were analyzed under HREC # 15/84 (Peter MacCallum Cancer Centre).

Note that full information on the approval of the study protocol must also be provided in the manuscript.
